# Breast Coil Optimization for Low Field MRI and Future MR-Guided Proton Therapy

**DOI:** 10.1109/TBME.2024.3520895

**Published:** 2025-04-22

**Authors:** Torben P.P. Hornung, Neha Koonjoo, Sheng Shen, Friderike K. Longarino, Kathryn E. Keenan, Susu Yan, Thomas R. Bortfeld, Matthew S. Rosen

**Affiliations:** Department of Physics, ETH Zürich,, Switzerland, also with the MGH/A.A. Martinos Center for Biomedical Imaging, USA, and also with the Department of Radiation Oncology, Radiation Biophysics Division, Massachusetts General Hospital, USA.; MGH/A.A. Martinos Center for Biomedical Imaging, USA, and also with the Harvard Medical School, USA.; MGH/A.A. Martinos Center for Biomedical Imaging, USA, and also with the Harvard Medical School, USA.; Department of Radiation Oncology, Radiation Biophysics Division, Massachusetts General Hospital, USA, also with the Clinical Cooperation Unit Translational Radiation Oncology, German Cancer Research Center (DKFZ), Germany, and also with the Department of Radiation Oncology, Heidelberg University Hospital, Germany.; Physical Measurement Laboratory, National Institute of Standards and Technology, USA.; Department of Radiation Oncology, Radiation Biophysics Division, Massachusetts General Hospital, USA, and also with the Harvard Medical School, USA.; Department of Radiation Oncology, Radiation Biophysics Division, Massachusetts General Hospital, USA, and also with the Harvard Medical School, USA.; MGH/A.A. Martinos Center for Biomedical Imaging, Charlestown, MA 02129 USA, also with the Department of Physics, Harvard University, Cambridge, MA 02138 USA, and also with the Harvard Medical School, Boston, MA 02115 USA

**Keywords:** MR breast imaging, proton therapy, ultra-low field, multi-objective optimization, pareto optimality formalism

## Abstract

**Objective:**

Magnetic resonance imaging of the breast and the chest wall imaging was performed at ultra-low field (ULF) using optimal radiofrequency (RF) coil designs and, with an eye toward future application in MR-guided proton therapy (MRgPT), simulation studies of a pencil proton beam trajectory in low and ultra-low magnetic fields were performed.

**Methods:**

The primary focus in this study is the RF coil design, which is approached as a multi-objective optimization (MOO) problem and effectively solved using a Pareto optimality formalism. Our coil winding pattern algorithm simultaneously optimizes the magnetic field homogeneity of the coil, the attainable MRI signal-to-noise ratio (SNR), and the magnetic field drop-off into the chest wall for improved OAR imaging. Five transmit/receive single channel breast-shaped RF coils were constructed, and 3D phantom MRI was performed at 6.5 mT. The RF magnetic field homogeneity across the imaging volume and SNR were analyzed among the different coils. *In vivo* breast imaging was performed for the first time at 6.5 mT. Monte Carlo simulations were implemented to study the influence of copper wire on a proton beam, as well as the beam deflection at various static field strengths.

**Results:**

Our MOO method was validated - the observed field homogeneity, the relative ratio of attainable SNRs for all five breast coils, and the MRI signal from the chest agree with the simulations. *In vivo* breast and chest wall images acquired at ULF reveal good breast tissue contrast and can also visualize the heart. Finally, the results of this work indicate that a field strength of ~50 mT with gradient strengths of ~10 mT/m to minimize proton beam deflection, and the ability to achieve a ~2 × 2 × 5 mm^3^ spatial resolution within few minutes are essential for making MRgPT clinically feasible.

**Conclusion:**

Our MOO method can be used to design breast coils that meet a wide range of imaging requirements.

## I. Introduction

Radiation therapy is a critical part of cancer care for at least 50% of all cancer patients [1]. For any given treatment area, there is a fine balance between delivering a radiation dose high enough to completely eradicate the tumor and minimizing the damage to the surrounding healthy tissue to reduce adverse effects. Among the different types of radiation therapy including brachytherapy, photon, and particle therapy (e.g., proton beam radiation), numerous studies have demonstrated that proton therapy (PT) can be more effective than photon therapy [2], [3], [4]. This is due to the compact nature of its dose distribution which is better confined to the targeted cancer tissue and can thus better spare healthy tissues than photon therapy [5]. With proton beam radiation therapy, there is a well-defined dose delineation with the highest dose delivered at the end of the beam (Bragg peak). This allows the tissue beyond the Bragg peak to be fully spared. Such a precise dose localization is particularly beneficial for protecting critical organs, known as organs-at-risk (OARs). To ensure that OARs do not move into the target volume during treatment, accurate imaging of the target structure and the OAR is essential [6]. In addition, imaging is currently used to validate the efficacy of PT, including the monitoring of the tumor size and potential radiation-induced side effects [7], [8].

A recent study shows that about 15%–50% of cancer patients could potentially benefit from PT with higher survival rates [4]. However, at present, only about 1% of radiation therapy is proton-based due to its high cost and its limited patient accessibility. Additionally a recurrently cited reason for the relatively slow adoption of PT in clinical settings is the lack of effective image guidance for PT [7], [9], [10]. Despite numerous projects focused on developing MR-guided PT (MRgPT) solutions, a fully integrated MRgPT system has not yet been implemented in clinical practice [10], [11], [12], [13]. To date, a variety of X-ray Computed Tomography (CT) guidance solutions are offered, however they mostly rely on 2D radiographs instead of volumetric images, and some are not capable of imaging the patient in the treatment position [14]. There is hence a need for advanced integrated (in-beam) image guidance for the success of PT.

Combining the optimal cancer targeting of PT with the superior imaging of MRI in a fully integrated system may enable the predicted benefits of PT to be realized. MRI is recognized as the gold standard imaging technique due to its superior soft tissue contrast, non-invasiveness, high spatial resolution, and high SNR, and would be an ideal modality for real-time imaging and motion management with adaptive radiation delivery based on the treatment plan [7]. In addition, MRI is not solely reserved for cases of near-adjacent tumor and OARs but is a valuable tool for ensuring precise tumor targeting, managing motion, and protecting healthy tissue throughout the treatment course [8].

Modelling and experimental investigations of the MRI interaction with radiotherapy protons have previously been addressed for various field strengths and proton energies [15], [16], [17]. A study based on actual measurements at field strength of 0.22 T with a static proton research beam line, demonstrated no visible beam-induced image degradation and that in-beam MR imaging is technically feasible. In addition, the work also stated the need to optimize RF coils that are transparent to proton beams [17]. Another study based on Monte Carlo simulations at various magnetic field strengths from 0 to 3 T showed a dose-dependent proton beam deflection greater than 10 cm at 1 T from its otherwise straight path [18]. The mutual interference of electromagnetic fields of the proton facility and the MR scanner can also compromise the beam and the image quality. To minimize this mutual interference, one solution would be to decrease the magnetic field strength, which has not yet been explored.

Low (<0.1 T) and ultra-low field (ULF) MRI (<0.01 T) has gained significant momentum in recent years [19], [20], [21]. The development of low-noise hardware and the integration of deep learning image reconstruction approaches have made MRI in the low field regime a very promising clinical imaging alternative [22], [23], [24]. A fully integrated system combining PT with a low field MRI scanner would require sufficient resolution for accurate tumor delineation, distinct tissue contrast with high signal-to-noise ratio (SNR) for precise anatomy monitoring and low/minimal beam deflection for high accuracy. All these imaging requirements shall then guide the design of RF coils, which is optimized for SNR, field homogeneity and spatial coverage for PT-relevant anatomies while ensuring compatibility with the PT treatment workflow.

We describe here our MRI-based breast imaging including organs-at-risk (OAR) in the chest wall at ULF using optimal RF coil designs. Given the possible use in future MR-guided PT, we performed an initial simulation study of the compatibility of a pencil beam trajectory with low and ultra-low magnetic fields. For breast imaging at ULF, an ideal RF coil should have high field homogeneity, produce high SNR images, and at the same time have high signal in the chest wall to visualize OAR. We describe the RF coil optimization as an inverse problem in which conflicting objectives need to be optimized simultaneously [25], [26]. With an aim of visualizing OARs, including the heart and lungs within the chest wall, we propose that RF coil design can be posed as a multi-objective optimization (MOO) problem [27] and can be solved successfully by using Pareto optimality formalism. Built on a proven RF coil optimization method [28], the coil winding pattern for this application is determined by optimizing three competing objectives: the magnetic field homogeneity of the coil over the volume of interest, the attainable SNR, and the spatial distribution of the magnetic field strength within the chest wall.

In the following, we describe in Section *A* the coil shape for breast imaging including OAR imaging in the chest wall and the physical constraints governing the groove pattern for the coil. The MOO method to identify optimal coil winding is presented in Section *B* and the algorithm is used to solve the optimal groove occupation pattern. The five single-channel transmit/receive breast-shaped RF coils for imaging at 6.5 mT which were built and tested are described in Section *C*. All five coils were evaluated via imaging, shown in Section *D*. Finally in Section *E*, we investigate the influence of the wire used for coil winding on the proton beam propagation using Monte Carlo-based simulations and determine the maximum magnetic field for a negligible proton beam deflection in a future hybrid system setup. By incorporating these simulations here, we offer a first indication of whether RF coils, beyond their proven imaging capabilities, might also meet the physical requirements necessary for integration into a future hybrid system, laying the groundwork for more detailed studies.

## II. Method

### A. Coil Geometry Selection and Groove Pattern Analysis

For breast imaging at ULF, close-fitting volume RF coils are the most suitable, due to their better filling factor and thus higher SNR [19], [29], [30]. Before optimizing the groove occupation pattern on the coil surface using the MOO method, the coil geometry was determined. Given the hemispherical/conical shape of the targeted imaging volume, two coils, one hemispherical and one conical, were selected [31], [32]. The most favorable coil geometry was determined by simulating the magnetic fields for both coil geometries using the Finite-Element-Method (FEM), as described in the next section, and with a slice-by-slice SNR analysis of phantom imaging. The MOO method was then applied to the selected coil geometry (Section *B*).

For a given coil shape, the groove pattern was determined based on three relevant parameters: 1) groove size (which affects the maximum wire occupation number in each groove), 2) the groove density on the surface (the distance between the individual grooves), and 3) the maximum radius of the grooves on the coil end plate. Consequently, the coil surface was tiled with many individual grooves (Fig. S1 in the Supplementary Material (SM)), and the magnetic field was simulated using FEM simulations. To evaluate the groove pattern, the $B_{1}$ field homogeneity within the central part of the breast volume and the $B_{1}$ drop-off in the chest wall were considered. The influence of the three groove pattern properties on the achievable $B_{1}$ field homogeneity in the breast and the $B_{1}$ field strength in the chest wall was analyzed using our optimization method described below.

### B. Multi-Objective Optimization Method

The workflow for this optimization is illustrated in Fig. 1. The optimization method consists of – FEM simulations (Ansys Maxwell, 2021, Ansys, Canonsburg, PA, USA) that were used to determine the magnetic field generated by the RF coil, Integer Linear Programming that was employed to calculate the resulting groove occupation pattern and the SNR for optimized coil parameters, and MOO algorithm based on the Pareto optimality formalism, which was used to select the optimal RF coils for breast imaging and OAR imaging in the chest wall.

#### 1) RF Coil Magnetic Field Calculation (Finite-Element-Method, FEM Simulation):

The total magnetic field generated by the coil can be calculated according to the superposition principle as the sum of the magnetic fields generated by the individual wire-turns in the grooves. As a first step, the magnetic field generated by a wire with unit current in each individual groove is determined using the FEM software. This is calculationally simple at 6.5 mT (i.e., a Larmor frequency of 276 kHz and a wavelength of order 1 km), since a simple direct current simulation can be used since high-frequency electromagnetic effects do not contribute. To evaluate the magnetic field in the simulation, a volume of interest (VOI) is first defined (Fig. S1c in the SM). The magnetic field strength is then evaluated in this VOI at 1000 evenly distributed target points. Since we want to determine the magnetic field within the breast and in the chest wall, the VOI not only fills the coil but also protrudes up to 4 cm beyond it at the large opening of the coil. Thus 800 target points were placed in the coil enclosed volume (breast volume) and 200 points in the chest wall region of the VOI.

The magnetic field at target point m produced by a unit current in groove n is denoted by the matrix element $A_{mn}$. The entries of matrix *A* are obtained from the FEM simulations and will be used to solve the integer linear programming problem below. By applying the superposition principle, this matrix can be used to compute the overall magnetic field at the target points within the VOI as follows (1):

(1) $$\overset{\to }{B}=A\cdot \vec{X},$$

where $X_{n}$ is the number of wire-turns in groove n, and $B_{m}$ is the resulting overall magnetic field at target point m. For each of the three coil geometries presented here, the magnetic field simulations were completed in about three hours using an Intel Core^™^ i7–8550U CPU @ 1.80 GHz, 1992 MHz, 4 core(s), 8 logical processor(s) with 16 GB RAM.

#### 2) Optimization Parameters (Integer Linear Programming):

The performance parameters of RF coils include SNR and the homogeneity of the magnetic field generated by the coil, while considering the quality factor of the coil (Q factor) and the resulting passband, as described in [28]. However, to meet OAR imaging requirements in PT, the optimization parameters include three criteria – the magnetic field homogeneity of the coil enclosed volume, the attainable SNR, and the signal strength in a VOI that protrudes 4 cm deep in the chest where the heart is seated (see Fig. S1c in the SM).

These optimization parameters were then reduced to an integer linear programming problem to determine the number of wire-turns in each coil groove $X_{n}$ element of $\overset{\to }{X}$ to generate a homogeneous magnetic field of a chosen field strength $B_{av}$ by using the least total wire length $S_{\text{tot}}$.

Using similar formalism as in [28], a set of (2) to (5) were adapted and solved using the intlinprog function in MAT-LAB R2022a (The MathWorks, Inc., Natick, MA, USA):

(2) $$Minimize\;:S_{\text{tot}}=\overset{\to }{S}\cdot \overset{\to }{X}$$

(3) $$\text{subject}\;\text{to}\;:\frac{1}{m_{\mathrm{tot}}}\sum_{m=1}^{m_{\mathrm{tot}}}\left|\left(\left(\sum_{n=1}^{n_{\mathrm{tot}}}A_{mn}\cdot X_{n}\right)-B_{av}\right)\right|\leq \delta_{1}\text{,}$$

(4) $$\begin{array}{l} \left|\frac{\sum_{n=1}^{n_{tot}}\left(A_{mn}\;\cdot \;X_{n}\right)\;-\;B_{av}}{B_{av}}\right|\times 100\leq \delta ,\\ m\in [1,\;2,\ldots ,800] \end{array}$$

(5) $$\begin{array}{l} \left|\frac{\sum_{n=1}^{n_{\mathrm{tot}}}\left(A_{mn}\;\cdot \;X_{n}\right)\;-\;B_{av}}{B_{av}}\right|\times 100\leq \delta_{\text{out}}\\ m\in [801,\;802,\ldots ,1000]\\ X_{n}\in \left[0,\;1,\;2,\ldots ,X_{max}\right]. \end{array}$$

This algorithmic approach makes it possible to maximize the SNR by determining the optimal groove occupation pattern to generate a constant, specified field strength $B_{av}$ in the coil enclosed volume. In (2), the component $S_{n}$ of $\overset{\to }{S}$ denotes the length of groove number n, and component $X_{m}$ of vector X is the occupation of groove m. The elements of $\overset{\to }{X}$ are only allowed to be integers, and their maximum values are limited by the cross sections of the corresponding grooves.

Equation (3) represents the condition where $\delta_{1}$ is the maximum allowed average absolute deviation of the magnetic field at the evaluation points, produced by the coil, from the selected field strength $B_{av}.\;n_{\mathrm{tot}}$ is the number of grooves on the coil surface (depends on coil geometry), and $m_{\mathrm{tot}}$ is the total number of evaluation points in the breast volume, equal to 800.

Another metric included in this optimization method is the homogeneity of the magnetic field (produced by the coil) in the breast volume to avoid outlier values. It is then quantified in (4), by the percentage deviation of the actually generated magnetic field at the target points from the desired field strength $B_{av}$ at each individual target point. δ is the maximum allowed percentage field deviation of the actual generated field from the desired field $B_{av}$ at the target points inside the coil enclosed volume (denoted by m).

Our third objective is to enable OAR imaging. Equation (5) represents the condition that the magnetic field strength at each target point outside the coil enclosed volume (i.e., within the chest wall) must be within a percentage deviation $\delta_{\text{out}}$ of $B_{av}$. Since the considered volume is outside the coil, the field strength decreases with increasing distance to the coil. Therefore, $\delta_{\text{out}}$ actually represents the maximum percentage drop-off in the chest wall. 200 target points were selected on this VOI where m∈[801,802,…,1000].

We note that SNR is defined as the ratio of the signal voltage to the noise voltage as described in [33]. Hence, the maximization of the SNR, as in [28], is reduced to the minimization of the coil resistance R as (7):

(6) $$SNR=\frac{\sqrt{2}\;\cdot \;B_{av}}{\sqrt{R}}.$$

By selecting different values for the target points inside the coil enclosed volume (δ) and for those outside the volume ($\delta_{\text{out}}$), it is possible to separate the magnetic field homogeneity condition inside the coil enclosed volume from the magnetic field strength analysis outside of it.

Solving the linear programming problem specifies the groove pattern of the coil for selected values of $B_{av},\;\delta_{1},\;\delta$ and $\delta_{\text{out}}$. This solution meets the requirements for the magnetic field generated by the coil with the shortest wire length, thereby maximizing the signal-to-noise ratio (SNR). However, solving the linear programming problem alone is not sufficient to achieve optimal coil design; an optimization of the parameters within the linear programming problem itself is also required. For a fixed target field strength $B_{av}$ and a fixed $\delta_{1}$, this optimization can be formulated as a multi-objective optimization (MOO) problem for the field homogeneity in the breast, the signal strength in the chest and the SNR.

#### 3) Mathematical Formalism of our Multi-Objective RF Coil Optimization (MOO) Algorithm:

##### Definition of the Solution Space:

For a fixed target field strength $B_{av}$ and a fixed $\delta_{1}$ the MOO algorithm defines the solution space, H comprising all pairs $\left(\delta ,\delta_{\text{out}}\right)$, where δ represents the percentage $B_{1}$ inhomogeneity within the enclosed coil volume (breast volume), and $\delta_{\text{out}}$ denotes the percentage $B_{1}$ field strength drop-off in the non-enclosed target volume (chest wall volume) as in (4) and (5) respectively (refer also to Fig. S1c in SM):

(7) $$H=\left\{\left(\delta ,\delta_{\text{out}}\right)|\delta ,\delta_{\text{out}}\in [0100]\right\}$$

##### Feasibility Boundary:

To establish the feasibility boundary, F, the algorithm iteratively reduces δ for each $\delta_{\text{out}}$ (in the linear programming problem described above) until no further feasible solution is found. F can be mathematically expressed as follows:

(8) $$F=\left\{\left(\delta ,\delta_{\text{out}}\right)\in H|\delta ={min}_{\left(\delta^{\prime },\delta_{\text{out}}\right)\text{is}\;\text{feasible}}\delta^{\prime },\delta_{\text{out}}\in [0100]\right\}$$

Connecting all those points gives the geometric feasibility boundary, as shown in Fig. S2a (in SM). Hence, for a selected coil geometry, which is comprised of both the coil shape and its groove pattern, this geometric feasibility boundary identifies all achievable $\left(\delta ,\delta_{\text{out}}\right)$-pairs. The solution space becomes:

(9) $$H^{\prime }=\left\{\left(\delta ,\delta_{\text{out}}\right)\in H|\exists \left(\delta^{\prime },\delta_{\text{out}}\right)\in F:\delta^{\prime }\leq \delta \right\}$$

##### Additional Constraints:

To prevent large inhomogeneities within the enclosed coil volume and low field strengths in the chest wall, the solution space, H is further constrained with the:

1. Maximum allowed percentage $B_{1}$ inhomogeneity level within the coil enclosed volume, $\delta_{\max}\;\left(\delta \leq \delta_{\text{max}}\right)$ and the
2. Maximum allowed percentage $B_{1}$ field strength drop-off in the chest wall volume, $\delta_{\text{out,max}}\left(\delta_{\text{out}}\leq \delta_{\text{out,}\;\text{max}}\right)$.

Thus, the resulting solution space, $H^{\prime \prime }$ is defined considering the chosen $\delta_{\text{max}},\;\delta_{\text{out,max}}$ and the feasibility limit, F (Fig. S2 in the SM):

(10) $$H^{\prime \prime }=\left\{\left(\delta ,\delta_{\text{out}}\right)\in H|\delta \leq \delta_{max},\delta_{\text{out}}\leq \delta_{\text{out},max},\exists \left(\delta^{\prime },\delta_{\text{out}}\right)\right.\;\left.\in F:\delta^{\prime }\leq \delta \right\}$$

For each point in the feasible solution space $H^{\prime \prime }$, the algorithm computes the maximum achievable SNR by solving the linear programming problem (see section above): Iteratively, the algorithm calculates the maximum achievable SNR for all $\left(\delta ,\delta_{\text{out}}\right)$ in the restricted space, by scanning $H^{\prime \prime }$ point by point in small increments of 0.1% (Fig. S2 b and c in SM).

##### Performance metric:

We define a weighted performance metric f, which combines contributions from $B_{1}$ inhomogeneity $\delta ,\;B_{1}$ field strength drop-off $\delta_{\text{out}}$ and $SNR\;\left(\delta ,\delta_{\text{out}}\right)=k$ with respective weights ${𝓌}_{1}$, ${𝓌}_{2}$ and ${𝓌}_{3}$:

(11) $$f\left(\delta ,\delta_{\text{out}},k\right)={𝓌}_{1}\left(1-\frac{\delta }{100}\right)+{𝓌}_{2}\left(1-\frac{\delta_{\text{out}}}{100}\right)+{𝓌}_{3}k$$

The performance metric f indicates the desirability of the coil attributes $\delta ,\;\delta_{\text{out}},\;k$ for specific imaging purposes. Thus, imaging purposes are incorporated into the optimization here through the weights ${𝓌}_{1}$, ${𝓌}_{2}$ and ${𝓌}_{3}$.

After introducing the solution space, constraining it with a physical feasibility boundary and two limits aligned with the imaging objectives, and defining a performance metric to evaluate the outcomes, two optimization methods are now presented to determine the optimal solution.

###### a) Maximizing the performance metric (solution method 1):

Direct maximization of the performance metric f offers an effective and straightforward solution approach for our MOO:

(12) $$\left(\delta^{*},\delta_{\text{out}}^{*},k^{\text{*}}\right)=\underset{\begin{array}{c} \left(\delta ,\delta_{\text{out}}\right)\;\in \;H^{\prime \prime }\\ k=\underset{\left(\delta ,\delta_{\text{out}}\right)}{\text{max}}\;SNR \end{array}}{\text{argmax}}f\left(\delta ,\delta_{\text{out}},\;k\right)$$

Here, $\left(\delta^{*},\delta_{\text{out}}^{*}\right)$ denotes the solution within $H^{\prime \prime }$ that maximizes the performance metric f with $k^{*}$ representing the maximum feasible SNR at $\left(\delta^{*},\delta_{\text{out}}^{*}\right)$. Of note, when setting ${𝓌}_{1}={𝓌}_{2}=0$, maximizing $f\left(\delta ,\delta_{\text{out}},k\right)$ simply yields the solution with the highest SNR in the restricted solution space (SNR is the dominant factor influencing image quality at ULF). We chose here to use a weighted performance metric over a weighted cost function for ULF MRI applications because it can be algebraically transformed into Iso-Desire lines, representing equally-valued solutions, used in the next section. However, in general, direct maximization of the performance metric yields multiple solutions that require further selection algorithms for determination.

###### b) Iso-SNR curves and Iso-Desire lines (solution method 2):

In more complex cases, where the SNR is subject to additional constraints – for instance, due to prior laboratory measurements that establish a minimum acceptable SNR, or when a specific target SNR level is defined or selected – the SNR level is fixed. The optimization objective then shifts to identifying the optimal solution along so-called iso-SNR curves.

##### Iso-SNR curves:

For different SNR levels k, all points ($\delta ,\delta_{\text{out}}$) within the restricted solution space $H^{\prime \prime }$ that achieve k as their maximum possible SNR level, can be described as:

(13) $$\left\{\left(\delta ,\delta_{\text{out}}\right)\in H^{\prime \prime }|\underset{\left(\delta ,\delta_{\text{out}}\right)}{max}\;SNR=k\right\}$$

When considering these solutions with the same SNR, we can disregard those solutions that have both a higher δ and a higher $\delta_{\text{out}}$ compared to another solution with the same SNR value. In other words, only those solutions are considered where all other solutions with the same SNR have either a greater δ or a greater $\delta_{\text{out}}$. These solutions are compiled into what we refer to as the iso-SNR curve of SNR level k:

(14) $$\begin{array}{ll} iso-SNR(k) & =\{\left(\delta ,\delta_{\text{out}}\right)\in H^{\prime \prime }|\underset{\left(\delta ,\delta_{\text{out}}\right)}{max}\\ SNR & =k,\forall \;\delta_{k}\delta \;\forall \;\delta_{\mathrm{out},\;k}\delta_{\text{out}}\;\text{with}\;SNR\left(\delta_{k},\delta_{\mathrm{out},\;k}\right)\\ \; & =k\;:\delta_{k}>\delta \vee \delta_{\mathrm{out},\;k}>\delta_{\text{out}}\} \end{array}$$

##### Trade-off relationship and Pareto optimality:

By Pareto optimality, for a given SNR level k, the corresponding iso-SNR curve delineates a set of nondominated and equally valid solutions in terms of homogeneity and signal strength (known as the Pareto fronts [34]), where no single solution can be improved in all objectives. For each such Pareto optimal solution there exists no other solution that is better in all objectives. Thus, for any solution on the Pareto front it is impossible to achieve a better value in one objective without at least one of the other objectives deteriorating.

The three critical quantities (SNR, $B_{1}$ inhomogeneity within the coil enclosed volume, and $B_{1}$ field strength drop-off in the chest wall) are competing objectives. $B_{1}$ inhomogeneity and $B_{1}$ field strength drop-off are directly related via the iso-SNR curves, while SNR is indirectly related, as increasing SNR shifts the iso-SNR curve to higher $B_{1}$ inhomogeneity and higher $B_{1}$ field strength drop-off levels. Thus, the optimal configuration of the coil depends on the specific imaging goals. To determine the optimal solution for a given imaging goal among the Pareto optimal solutions along the iso-SNR curve, we introduce the concept of iso-Desire lines, also known as indifference curves [35] (Fig. S2d).

##### Optimal Configuration and Iso-Desire Lines:

The optimal configuration ($\delta^{*},\delta_{\text{out}}^{*}$) for a chosen iso-SNR curve is the point on the curve with the highest performance metric f. Geometrically, this can be illustrated as follows: given that the optimal SNR level is $k^{*}$, it can be shown from (11), that all points in the solution space achieving a fixed performance level $f\left(\delta ,\delta_{\text{out}},k^{*}\right)=t$ are given by the following line, which we refer to as the iso-Desire line:

(15) $$\delta =100\cdot \left(1+\frac{{𝓌}_{2}}{{𝓌}_{1}}+\frac{{𝓌}_{3}}{{𝓌}_{1}}\cdot k^{*}-\frac{t}{{𝓌}_{1}}\right)-\frac{{𝓌}_{2}}{{𝓌}_{1}}\cdot \delta_{\text{out}}$$

Thus, iso-Desire lines quantify imaging goals by representing all solution pairs of $B_{1}$ inhomogeneity within the coil enclosed volume and $B_{1}$ field strength drop-off in the chest wall that achieve, for a fixed SNR, the same level of overall performance for specific imaging goals. To note, an increase in performance level t corresponds to an inward shift of the iso-Desire line.

The derivatives of these iso-Desire curves represent the willingness to sacrifice $B_{1}$ inhomogeneity within the coil enclosed volume in order to gain increased $B_{1}$ field strength within the chest wall. Thus, the slopes of the iso-Desire lines incorporate individual imaging aims into the optimization process of the optimal coil. Within our narrowly restricted solution space, this marginal rate of substitution [35] remains constant. For a given SNR, the optimal solution is where the iso-SNR curve intersects the iso-Desire line with the highest performance level t. In the case of a convex iso-SNR curve, this optimal solution is defined by the tangential intersection of the iso-Desire line with the iso-SNR curve. For instance, a steep iso-Desire curve aims at achieving a strong signal in the non-enclosed target volume, which might be necessary to see OAR such as the heart (Fig. S2c in the SM).

The code used in this study is publicly available on Code Ocean at https://codeocean.com/capsule/2107664/tree/v1, with a DOI of https://doi.org/10.24433/CO.2675877.v1. This capsule contains the necessary executable scripts to illustrate the MOO analysis presented in this manuscript. For any queries regarding code access or usage, please contact the corresponding author.

### C. Design and Construction of 5 Different Breast-Shaped RF Coils

Fig. 2 illustrates the five close fitting transmit/receive RF coils with different shapes and/or sizes for OAR and breast imaging. The properties of each of the coils are summarized in Table I. All coils were designed using CAD software (Fusion 360, Autodesk, USA). The five coils were 3D printed, wired using stranded Litz wire (Type 15/39/42, New England Wire Technologies, NH, USA), tuned to 276.18 kHz and matched to 50 ohms with a return loss of at least −40 dB.

The ratio of the loaded to unloaded Q factor for all the breast coils is approximately 1. For instance, Coil 2 had an unloaded Q factor of 87.5 and a loaded Q factor of 88 (as measured on a Keysight N9913A FieldFox RF Analyzer). The unloaded Q factor of the coils ranged from 78 to 90 as reported in Table I. During frequency encoding, the readout gradient imposes a minimum bandwidth across the field-of-view (FOV). This may exceed the receive coil bandwidth if the coil Q is too high. To ensure the entire FOV in the readout direction is not attenuated, the passband of RF coil must be not narrower than the readout bandwidth. According to the relationship between SNR and Q factor, the lower the Q factor, the lower the SNR of RF coil. Ideally, the Q factor of a RF coil should be maintained as high as possible provided it meets the readout bandwidth constraint. In this work, the Q factor of the RF coil was adjusted by adding a resistor in series with the RF coil.

The typical power to generate a 300 *μ*s 90° pulse with this coil on our system at 6.5 mT is 50 mW. This corresponds to a voltage across the coil of 5 volts. The Litz wire used for coil winding, was a custom-built insulated Fluorinated Ethylene Propylene (FEP) wire with a breakdown of this insulation to ground at 8 KV. The wire itself is rated for use up to 500 V, and arcing does not occur in our system.

A brief description of the five RF coils is given below:

1. Small Hemispherical Coil: The shape of Coil 1 was chosen as a simple geometry which can accommodate a typical female breast. The coil has an outer diameter of 14 cm (Fig. 2(a)).
2. Small Conical Coils: Based on promising study results at higher field strengths [31], two conical breast coils were also designed. The size of the conical coil was adapted to the most common female breast size in the US [36]. The coil height is 10 cm, its outer diameter at the end plate is 14 cm, and its outer diameter at the peak is 4 cm. The first small conical coil, Coil 2 (small unoptimized conical breast coil), had one wire-turn in each groove (Fig. 2(b)). The second coil, Coil 3 (small optimized conical breast coil), was optimized for a better $B_{1}$ field homogeneity in the breast and a deeper view into the chest wall compared to Coil 2 (Fig. 2(c)).
3. Large Conical Coils: To enable imaging of larger breasts, we designed large conical coils with the same height as the small conical coils and the same diameter at the peak, but the outer diameter at the end plate was 19 cm. Two large conical coils were built, each optimized with a different aim. As seen in Fig. 2(d), a large conical coil was optimized with the aim of enabling the deepest possible view into the chest wall while achieving an acceptable SNR and an acceptable $B_{1}$ homogeneity in the breast, referred as Coil 4. The second large conical coil, Coil 5, was optimized for the best possible imaging of the breast (Fig. 2(e)).

### D. Imaging

Experimental evaluation of the coil optimization method was carried out through imaging using a previously described custom built 6.5 mT MRI system (Larmor frequency of 276.18 kHz) [29]. The magnetic field of the MRI scanner is generated by a high-homogeneity electromagnet with biplanar imaging gradients in all 3 spatial directions with 1 mT/m peak gradient amplitude. In order to carry out the experiments with the best possible $B_{0}$ homogeneity, the coils in all experimental setups were placed at the isocenter of the magnet. In order to position the coils at the isocenter of the scanner, a height-adjustable board was used for phantoms and for human imaging (Fig. S3 a and b in the SM). The breast coils were mounted on a custom-built support system, which was attached to the height-adjustable board (Fig. S3 a and b in the SM).

#### 1) Phantom Imaging:

To evaluate the imaging performance, two phantoms were constructed, one hemispherical and one conical (Fig. S3 c and d in the SM). These phantoms were filled with 0.625 mg/ml CuSO_4_ solution in de-ionized water, aiming for a nominal T_1_ relaxation time of 200 ms. Unlike in MRI phantoms used at high field, the additional salt to approximate dielectric body loading was not added because at our Larmor frequency of 276 kHz, we observe no body loading. Indeed, these coils have a measured unloaded Q to loaded Q ratio close to 1. The imaging experiments were designed to evaluate several characteristics of coil quality: the homogeneity of the $B_{1}$ field in the chest volume, the signal strength 4 cm within the chest wall, the image SNR, and the attainable image resolution. To compare these characteristics of the different coils with each other (and with the results of the optimization algorithm), we performed phantom imaging using the 5 breast coils. Imaging was performed using a 3D bSSFP pulse sequence with the following parameters: TE/TR = 13/26 ms, in-plane matrix size = 64 × 75, number of slices = 20 (for hemispherical coil) and 25 (for conical coils); slice orientation = coronal (see Fig. S3d in the SM); voxel size = 3.5 mm × 2.5 mm × 5.5 mm, NA = 20, acquisition time = 14 minutes and 15 seconds.

The SNR and the coefficient of variation (CoV) were evaluated for the bSSFP phantom images. SNR was obtained by calculating the mean signal amplitude in a ROI divided by the standard deviation of the noise in a region considered background (see Fig. S4 in the SM). The SNR was measured for each slice. Each phantom imaging experiment was repeated 10 times and the mean SNR and its standard deviation were plotted for each slice. The CoV defined as standard deviation over the mean of the signal intensity inside the ROI was evaluated using bSSFP phantom images and magnetic field maps.

#### 2) In vivo Imaging:

*In vivo* breast imaging was performed on two healthy volunteer human subjects under IRB approval at our institution using the same 3D bSSFP sequence as in phantom imaging. Imaging was performed on one healthy female volunteer and one male volunteer in sitting position (Fig. S3b in the SM) using Coil 4 and Coil 5. Sagittal slices were acquired (Fig. S3d in the SM). The imaging parameters were as described above with a spatial resolution = 3.5 mm × 2.5 mm × 7 mm, matrix size = 64 × 75 × 15, NA = 40, and a scan time = 16 minutes and 45 seconds.

The signal depth achieved *in vivo* using both RF coils was measured. An analysis of the heart-skin distance of the two volunteers was measured using images from Coil 4. The distance from the heart to the skin (male volunteer) or to the breast pad (female volunteer) along the midclavicular line and along the midsternal line was determined using 6 repeated scans.

### E. Monte Carlo Simulations of a Low/Ultra-Low MRI System Coupled With a Proton Beam

Two experimental setups were implemented to assess the deflection of a proton beam as it passes through layers of copper wire (arranged as in the different RF coil designs) and to study the influence of the magnetic field strength on the proton beam trajectory (Fig. S5 in the SM).

An open source, object-oriented simulation toolkit written in C++ called GEANT4 (for GEometry ANd Tracking 4), developed by the European Organization for Nuclear Research collaboration, CERN (Release 11.0) was used [37]. In the first experimental setup, a generic proton pencil beam was defined to cross different numbers of wire layers, and the change in projected range was determined in a water tank placed behind it [38]. In the second simulation, a generic proton pencil beam passing through a homogenous magnetic field region of varying field strengths was defined. A water tank was placed at the center of the homogeneous magnetic field and the Bragg peak deflection was determined. Of note, the use of the water tank ensures geometric invariance, is independent of precise contours of a chosen breast shape, and is common practice for depth-dose curve measurements in experiments.

#### 1) Wire Influence on the Proton Beam:

A 50 cm × 50 cm × 50 cm large water tank phantom was placed in the middle of the simulated world (Fig. S5a in the SM). The ionization potential of liquid water was set to 75 eV [39]. To simulate a single wire, a copper cuboid with a thickness of 0.55 mm and a cross-section of 0.55 mm × 500 mm was placed at a central height at 5 mm from the water tank (i.e., the distance that the wire of our coils has from the skin). Finally, monoenergetic protons with an energy of either 150 MeV or 200 MeV were generated 10 cm from the copper cuboid. The momentum of the protons was directed towards the block. A vacuum was created in the simulated world and the effect of the copper wire on the protons was evaluated. In each simulation run, the energy and the dose of the protons in the water phantom were determined in longitudinal and lateral direction to the beam.

Given that the RF coils can accommodate up to three layers of wire in the chest area (Fig. S1d in the SM), three simulation setups with copper thicknesses of 0.55 mm, 1.1 mm, and 1.65 mm were chosen. This thickness corresponds precisely to that measured of the Litz wire we used. For each simulation setup, 100000 simulation runs were conducted to minimize statistical fluctuations while still achieving a reasonable simulation time.

To ensure that the important properties of the proton interactions in matter are simulated correctly, the pre-implemented reference physics list was selected [40].We chose the QGSP_BIC_EMY physics list for this [40], [41]. Class G4PVReplica was used to subdivide the water tank into 500 slices of 1 mm thickness perpendicular to the initial proton beam direction. To determine the energy and the dose, two scoring meshes (one longitudinal and one perpendicular to the beam) were implemented using class G4ScoringManager. For the generation of protons, the class G4ParticleGun was employed. For the wire, the copper properties implemented in the NIST GEANT4 material database were used [42]. We defined the properties of the vacuum in the Detector Construction class ourselves, using the galactic vacuum predefined in GEANT4 with a temperature of 293.15 K, a pressure of 10^−19^ Pa and a density of 10^−19^ g.

#### 2) Proton Beam Deflection in the Magnetic Field:

A 2 m × 2 m × 1 m large homogeneous magnetic field was generated in the middle of the simulated world (Fig. S5b in the SM). This homogeneous magnetic field is intended to represent a simplified version of the MRI device. The water tank phantom as described above was placed at the isocenter of the simplified MRI system. Monoenergetic protons with an energy of either 150 MeV or 200 MeV were generated at 100 cm from the isocenter of the simulated MRI system. The momentum of the protons was directed towards the water phantom such that the initial velocity vector was perpendicular to the $B_{0}$ direction of the MRI device. The entire assembly was placed in vacuum to ensure that the deflections of the protons were caused solely by the deflection in the magnetic field. In each simulation run, the energy and the dose of the protons in the water phantom were determined in longitudinal and lateral direction to the beam.

The first simulation runs without a magnetic field were carried out to obtain a reference position of the Bragg peak in the water phantom. After every 100000 simulation runs, the magnetic field strength of the MRI system was successively increased. The following increasing field strengths were used: 6.5 mT, 20 mT, 40 mT, 60 mT, 64 mT, 80 mT, 100 mT and 500 mT. The 64 mT-simulation runs match the field strength of a commercial low field MRI system (Hyperfine, Inc) [22].

Eight simulation setups were selected and for each setup 100000 simulation runs were conducted. In order to simulate proton propagation in the magnetic field of the MRI scanner, we included the G4FieldManager class and the G4TransportationManager class. A classical fourth-order Runge-Kutta stepper was implemented to check the accuracy of the proton propagation in the magnetic field. The same QGSP_BIC_EMY physics list was used [40], [41]. The water tank was subdivided using the G4PVReplica class into 5000 slices at 0.1 mm thickness. The energy and dose distribution were determined by the G4ScoringManager class, for which two scoring meshes (one longitudinal and one perpendicular to the initial beam direction) were defined. The G4ParticleGun class was used to generate the protons. The vacuum properties from the wire influence simulation were used.

## III. Results

### A. Coil Shape Analysis

The two chosen coil geometries – the hemispherical (Coil 1) and the conical (Coil 2) breast RF coils were designed based on the same outer end plate diameter of 14 cm. This physical constraint resulted in a ~10% smaller volume for Coil 1 compared to Coil 2. Defined as the percentage of the female population with breasts smaller than the respective coil volumes, the volume percentiles of Coil 1 and Coil 2 are 79% and 86% respectively. Based on the calculated percentiles [43], it should be noted that the effective percentile in the Coil 2 is slightly smaller than the calculated one. This discrepancy arises because the cone does not precisely match the natural shape of a breast, making it difficult to fully occupy the tip of the cone.

The baseline for Coil 1 and Coil 2 is that they are both unoptimized. Simulations reveal that Coil 1 and Coil 2 exhibited approximately the same level of $B_{1}$ inhomogeneity, expressed as the percentage CoV in the breast volume (± 0.33 for Coil 1, ± 0.31 for Coil 2) (Table II). The percentage of signal strength that dropped off at 4 cm inside the chest wall was higher for Coil 1 (81%) than for Coil 2 (78%).

Based on the imaging results shown in Fig. 3(a) and (b), the two coils showed approximately the same level of homogeneity in the direction parallel to the main coil axis. For an acquisition time of 14 minutes 15 seconds, a maximum SNR of 110 was measured in the images of the conical coil, but in those of the hemispherical coil, only a maximum SNR of 90, which confirmed the simulation predictions (Fig. 3(c)–(e)). The CoV measured in the breast volume of the bSSFP phantom images were similar for both coils (± 0.56 for Coil 1, ± 0.60 for Coil 2) (Table II). Overall, based on our simulations and experimental comparisons, the specific linear arrangement of the groove pattern and spacing of the conical-shaped coil provided a geometrical filling-factor advantage over the hemispherical-shaped coil resulting in a higher SNR.

### B. Groove Pattern Analysis

The influence of the groove pattern on the physical coil properties was evaluated on Coil 2. Since it is intended to achieve both good image quality in the breast volume and in the chest wall, the selected coil geometry was divided into two parts: a conical chest part and an extended coil end plate near the chest wall, as seen in Fig. S1 (in the SM). The influence on the achievable $B_{1}$ field homogeneity in the breast and the chest wall of the groove size, the groove density on the surface, and the maximum radius of the grooves on the coil end plate is shown in Fig. 4.

The maximum permissible number of wire-turns per groove was varied for the breast part of the coil (as seen in Fig. 4(a)) to analyze the influence of the groove size on the solution space. A higher occupation limit leads to a higher feasible homogeneity level in the breast. However, the effect saturates with a maximum groove occupation of 6 for a single groove 2.5 mm broad and 3.5 mm deep (Fig. S1d in the SM).

As observed in Fig. 4(b), the distance between the grooves in the breast part of the coil also had an influence on the feasible $B_{1}$ field homogeneity. A higher groove density can generate more homogeneous magnetic fields in the breast volume. Hence, the groove distances in the breast part of the coils were chosen to be as small as possible for all coils.

In addition, the influence on the solution space of the groove density at the coil end plate was analyzed (Fig. 4(c)). The distance between the grooves (within a reasonable range) does not have a large influence. On the end plate of the coils, the maximum wire occupation of a single groove (3.5 mm broad and 6 mm deep) is set to 10 to ensure that the inductance and, consequently, the Q factor does not increase excessively. However, the addition of concentric grooves with larger radii increases the solution space (Fig. 4(d)). Hence, the maximum groove radius at the coil end plate was selected to be as large as possible. To what extent the resulting new solutions are optimal in terms of image quality can be determined by solving the introduced MOO problem. In particular, the SNR of the coil drops significantly with grooves of larger radii.

### C. Solution Spaces for the Introduced Optimization Method for the Small and Large Conical Coils

The results described so far clearly inferred the choice of a conical-shaped coil and set the physical limits for groove patterns of the coils. They both served as the pre-requisites for solving the MOO problem.

#### 1) Small conical coils – Coil 2 and Coil 3:

A restricted solution space was obtained for the small conical breast coil, where the maximum permitted $B_{1}$ inhomogeneity in the breast, $\delta_{\text{max}}$ and the maximum permitted $B_{1}$ drop-off in the chest wall, $\delta_{\text{out,max}}$ were chosen to be ±10% and 60% respectively. As seen in Fig. S6 (in the SM), the simulations predicted that Coil 3 would have a homogeneous signal in the breast volume and would produce a stronger signal in the chest wall region with the trade-off of a lower SNR compared to Coil 2.

Phantom imaging using Coil 3 (Fig. 5(a)) shows a more homogeneous SNR across the slices compared to Coil 2 (Fig. 3(b)). The groove occupation pattern for each coil is listed in Table II. The signal amplitude, noise level and SNR plots are given in Fig. 5(b)–(d), respectively, and validate the simulated data. The noise level in both coils were comparable. The signal amplitude and SNR in Coil 3 was similar across all slices, and a stronger signal was measured in the chest wall region (between Slices 1 and 7) compared to Coil 2. As expected, the SNR trade-off is clearly visible. For the slices in the breast volume, the SNR values increase gradually across slices starting from 20 and reaching a maximum of 110 for Coil 2. For Coil 3 with the optimized groove occupation pattern, the SNR values varied between 40 and 60 across slices in the entire breast volume with an overall higher homogeneity and deeper view in the chest wall region as evidenced by a reduction of CoV by ~54% (from 0.597 to 0.277) for the bSSFP signal intensity (Table II). The reduction of CoV for the bSSFP signal intensity validates the simulations, which resulted in a CoV reduction of 55% (from ±0.31 to ±0.14).

Due to the SNR trade-off observed with Coil 3, we evaluated SNR at 3 different Q factors of 65, 84 and 145 by varying the added resistance in the LC circuit. As seen in Fig. 5(e)–(g), the SNR plots show that with increasing Q factor, the SNR also increases. However, at a Q factor of 145, the imaging bandwidth is too narrow (Fig. S7 in the SM). When the Q of the coil is too high, the passband of the coil is smaller than the readout bandwidth, and it results in a hyper-intense center band across all the slices. To avoid this issue, we adjust the Q of the coil to be compatible with the readout bandwidth. As a result, a Q factor of 84 was chosen for Coil 3.

#### 2) Large conical coils – Coil 4 and Coil 5:

Two optimized solution spaces were obtained for the two large conical breast coils. The solution space for Coil 4, was restricted by physically unattainable $\left(\delta ,\delta_{\text{out}}\right)$-pairs, the maximum permitted $B_{1}$ inhomogeneity in the breast, $\delta_{\text{max}}$ at ±10% and the maximum permitted $B_{1}$ drop-off in the chest wall, $\delta_{\text{out,max}}$ at 40% (Fig. 6(a)). For Coil 5, $\delta_{\text{max}}$ and $\delta_{\text{out,max}}$ were chosen to be ±10% and 80% respectively. The iso-SNR curve is falling (negative first derivative) but with a positive second derivative (Fig. 6(b)). The falling character of the curve corresponds to a trade-off between the $B_{1}$ homogeneity in the coil enclosed volume (breast volume) and the $B_{1}$ field strength outside the coil enclosed volume and within the chest wall.

The positive second derivative could be described as diminishing marginal returns. If the magnetic field in the breast is not very homogeneous, one does not have to sacrifice much signal in the chest wall to gain a lot of homogeneity. However, the more homogeneous the magnetic field in the breast already is, the more signal in the chest wall one must sacrifice to achieve the same increase. The further outwards (to the right) the iso-SNR curve is selected, the higher the SNR, but at the same time, the worse the field homogeneity in the breast volume and/or the field strength in the chest wall become. Hence, to find the sweet spot of $B_{1}$ homogeneity, the field strength within the chest wall, and SNR, two different objectives were chosen: one for OAR imaging (Coil 4) and one for breast volume imaging (Coil 5).

Two iso-Desire lines of different slopes were used. A steep iso-Desire line was chosen for OAR imaging with ${𝓌}_{2}/{𝓌}_{1}=2$. The latter was tangential to the iso-SNR curve in the upper left part of the solution space, thus resulting in a coil with a strong signal in the chest and a relatively low homogeneity level in the breast volume (Fig. 6(b)). A flat iso-Desire line was used for breast imaging $\left({𝓌}_{2}/{𝓌}_{1}=0.5\right)$. This iso-Desire line was tangential to the iso-SNR curve in the lower right part of the solution space, thus resulting in a high level of homogeneity in the breast volume (Fig. 6(c)). For a higher homogeneity in the breast with the same wire length, we traded off the field strength in the chest.

With the resulting groove occupation patterns (Table II), magnetic field strength was simulated for Coil 4 and Coil 5. As shown in Fig. S8 (in the SM), both coils produced homogeneous $B_{1}$ fields in the breast volume. The CoV values were ±0.17 for Coil 4 and ±0.16 for Coil 5 respectively. The simulated $B_{1}$ maps also showed that Coil 4, optimized for OAR imaging, generated a stronger field in the chest.

These simulation results of the two chosen Pareto optimal solutions were confirmed through phantom imaging. The images acquired with both optimized coils are very homogeneous and have a better signal intensity in the chest wall part of the phantom (Fig. 7(a) and (b)) compared to the images acquired with Coil 2 (Fig. 3(b)). Both coils enabled clear visualization of the first 7 slices, which is a depth of about 4 cm into chest wall (Fig. S1c and S3d in the SM). The maximum SNR in the chest region using Coil 4 was 48, which was significantly higher than the SNR in the same region with Coil 5 of 40 (Fig. 7(c)–(e)). The noise level in Coil 5 was slightly higher than that of Coil 4 (Fig. 7(d)). The SNR level in the breast region is similar in both Coil 4 and Coil 5, as evidenced by CoV values measured in the breast volume of 0.22 for Coil 4 and 0.21 for Coil 5 respectively.

### D. In Vivo Breast Imaging

*In vivo* imaging was performed to validate the MOO algorithm. The images acquired with a female volunteer, shown in Fig. 8, extend from the midsternal line over the midclavicular line to the lateral end of the chest. We note that the volunteer did not experience any discomfort during the exam and her breast fit naturally in the conical-shaped RF coil without any compression. The same region was visualized on a male volunteer (Fig. S9 in the SM). In both volunteers, the musculus pectoralis major, the ribs and the intercostal muscles, the anterior region of the lungs and the heart can be observed. As seen in Fig. 8, Coil 4 can obtain signal farther in the chest compared to Coil 5. An analysis of the visual depth range into the chest wall showed that a 10.1 cm depth was achieved with Coil 4 and a 7.8 cm depth with Coil 5. Both depth ranges are significantly larger than those of the small conical coils, owing to the larger coil diameters.

In breast cancer PT, the heart-skin distance is a crucial measurement in evaluating the OAR exposure, the target dose coverage, and the normal tissue compatibility probability value [44]. The heart-skin distance of the male volunteer while sitting was measured at 7.3 ± 1.2 cm (subcostal) and 3.8 ± 0.9 cm (parasternal). In the case of the female volunteer, the distances were 5.9 ± 1.1 cm (subcostal) and 3.1 ± 0.8 cm (parasternal).

### E. Monte Carlo Simulations

With an increasing number of wire layers, the projected range of the protons in the water tank decreases by an additional 3.2 ± 0.1 mm per layer of wire for a proton energy level of 150 MeV and 3.4 ± 0.1 mm for 200 MeV (Fig. 9(a)). This linearity can be explained by the fact that the energy loss of the protons in the copper wire is so low that the mass stopping power of the copper hardly changes with each additional layer. With an increasing number of penetrated wire layers, the lateral spread of the proton beam also increases. However, this increase is only of the order of 0.1 mm.

In the second simulation the magnetic field strength was varied, and the proton beam deflection was assessed. For each magnetic field strength, the exact position of the maximum dose deposition in the water tank was determined. This was done by adding up the dose distributions of the individual simulation runs and then determining the volume element with the maximum overall dose. To calculate the absolute shift to the reference position determined at B = 0 T, the length of the deflection vector, $\overset{\to }{D}$ was then simply calculated as (16):

(16) $$|\overset{\to }{D}|=\sqrt{D_{x}^{2}+D_{y}^{2}+D_{z}^{2}}$$

The deviations from the reference position for the different magnetic field strengths of the simulated MRI systems are plotted in Fig. 9(b). As expected, the total Bragg peak deflection increased with increasing magnetic field strength and increased for higher proton energy level [45]. According to the simulation, the 6.5 mT system results in a submillimeter-level shift. Of note, the total Bragg peak deflection of 10 mm at 0.5 T corroborates simulations published in [46] where an initial 200 MeV proton beam was used.

## IV. Discussion

The success of PT relies on its high conformal target dose distribution compared to radiotherapy with photons, to better spare OARs [47] and to minimize dose to non-target tissues, particularly in areas where long-term side effects are a concern [48]. In particular for breast cancer, the potential for reduced radiation exposure to the heart, lungs, and contralateral breast is a compelling reason to consider PT, even if OARs are unlikely to move into the treatment volume. To date, MR guided proton therapy has been focused at higher field strengths (>0.2 T) [13], [17], [41] where the main challenge is to minimize the mutual interactions between both systems. This study explored the technical feasibility of designing and optimizing RF coils for OAR and breast imaging at ULF, and it also investigated through simulations the impact of the magnetic field on the proton beam trajectory.

To our knowledge, for the first time, a multi-objective RF coil optimization method was implemented and validated with the ability to optimize coils of any shape and with any required imaging goal. The MOO algorithm simultaneously optimizes the SNR of the coil, the homogeneity of the magnetic field generated by the coil, and the received signal strength over a selected volume. Here we applied the algorithm to breast imaging, where tracking the position of the heart and imaging the target volume during PT treatment are crucial to deliver accurate dose distribution [49]. However, in the context of MRgPT, this algorithm could also be applied to other anatomical structures, for instance the brain or the thyroid. Coils of different shapes and different sizes, unoptimized and optimized for different imaging goals, were built, and the functionality of the proposed method was validated by phantom imaging on a 6.5 mT MRI system. The MOO problem presented can be solved using various methods; the procedure presented here is a particularly straightforward and intuitive approach. Future research should explore the application of different solution methods and compare the efficiency and outcomes of these procedures.

Phantom imaging was performed with a 3D bSSFP sequence for the five breast coils. The different image qualities achieved with the differently optimized coils corroborated the respective optimization aims. The images acquired with the optimized coils demonstrated a higher SNR homogeneity with a significant CoV reduction of >50% and a 4 cm deeper view into the chest wall than those images acquired with the unoptimized coils. Both the SNR trade-off observed in the imaging results and the effect of increasing the Q factor on the imaging bandwidth corroborated the recently published paper by Shen et al. [28]. Additionally, the choice of testing two coil shapes is mainly based on existing literature, showing that the conical shape fits a wide range of breast sizes while maintaining a good filling factor [31]. Since the filling factor plays an important role in maximizing SNR, the coil shape should also be included in future optimization algorithms.

The groove occupation pattern analysis provides further understanding that breast coil designs should incorporate a large maximum number of wires in the grooves, a high groove density in the breast part of the coil and a large maximum groove radius on the coil end plate.

*In vivo* breast and chest wall imaging was performed on both a male and female volunteer in sitting position at ULF, achieving reasonable spatial and temporal resolution. The SNR-efficient 3D bSSFP imaging sequence was used for image acquisition. In the *in vivo* images, the internal structures of the female breast, the musculus pectoralis major, ribs, intercostal muscles, as well as parts of the lungs and the cardiac wall could be recognized. This experiment also validated the proposed MOO algorithm, showing a 10 cm deep view into the chest and a homogeneous signal across the slices in the breast volume with the chest-optimized conical coil. The heart-skin distance of the volunteers agrees with recent studies that found a mean parasternal distance of 3.2 cm and a mean paracostal distance of 7.1 cm (no gender distinction was made) [50]. Male chest wall imaging was as useful as female chest wall imaging for validating our optimization method and coil winding patterns. Although the breast RF coil was not specifically designed for male chest wall imaging, we were able to demonstrate its applicability in this context. The main drawback of the *in vivo* image quality was the increased noise level and therefore the need to increase the number of averages for a better SNR. Further *in vivo* experiments are required to validate the patient’s comfort and imaging requirements. Additionally, different coil shape designs or multichannel RF coils can also be envisioned and optimized for low field breast and chest wall imaging [19], [51], [52]. Further studies of the influence of the patient position (prone vs. upright) on the imaging will be performed to understand the impact of gravity, the detection of OAR and the design of the hybrid MRI-guided proton therapy system. Minor adaptations to the RF coil design may be needed for coverage, positioning, and patient comfort based on imaging objectives and patient positioning in a low-field MRgPT system; however, the fundamental principles of the coil’s performance—such as its resonance frequency, B1 field distribution, and sensitivity—would remain consistent.

PT has very specific image quality requirements. For image guidance in particular, the spatial and temporal resolution of the images must be high enough to resolve target structures in a timely manner and OAR must be visible. Hence, to meet the clinical treatment plan, the spatial and temporal resolution should be better than the results presented here. In particular, spatial resolution should be 3D with a voxel size of at least 2 mm × 2 mm × 5 mm and a scan time of a few minutes. To achieve this voxel size at 6.5 mT, we would require ~3.5 times more averages, leading to a scan time of over one hour.

Furthermore, the static magnetic field of the MRI component of an envisioned hybrid system will have an impact on the proton beam trajectory. We aim for a targeting accuracy of 1 mm based on the broadening of the Bragg peak due to range straggling and multiple Coulomb scattering. Specifically, the range straggling amounts to approximately 1% of the range, i.e., 1 mm for a typical range of 10 cm [53]. As shown in Monte Carlo simulations, at 6.5 mT, the deflection of the protons is negligible, which thereby raises the question: to what extent can the magnetic field strength be increased? For field strengths below 50 mT, a shift of the Bragg peaks in the sub-millimeter range could be determined. A field strength of 100 mT led to a shift of 2 mm (for monoenergetic 200 MeV protons). In order to obtain the best possible image quality, under the condition that the deflection of the protons is negligible, the hybrid system must be designed within the low field regime (between 0.01 T to 0.1 T). Of note, beam deflection correction at higher field strengths (>0.1 T) is possible, however it still remains experimental [17], [54], [55].

The lateral spreading of the proton beam through several layers of wire and the shift in the Bragg peak location were in the sub-millimeter range. For each additional layer of wire in the path of the protons, the penetration depth of the proton beam decreased by a further 3 mm. However, since large areas of the coils are wire-free, this problem should be solvable in practice by including the coil geometry in the therapy optimization and planning. In this study, the effect of the static magnetic field and the effect of copper wires of an RF coil on the proton beam delivery were considered. However, future work shall also investigate how time-varying gradient fields can also impact the proton beam.

To date, several low field MRI systems (prototypes [56], [57], [58], [59] and one commercial [22], [60]) exist already and have proven to acquire diagnostic-grade images [21]. For instance, the 64 mT Hyperfine scanner is able to acquire T_1_-weighted images in the human head in less than 5 minutes with spatial resolution of 1.5 mm × 1.5 mm × 5 mm [22]. Another example, a 50.9 mT permanent magnet can acquire images of hemorrhagic stroke in approximately 3 minutes of scan time [61]. With these constant advances made in low field MRI technology, it is possible to envision a hybrid MRI guided proton therapy system that will be less expensive and accessible to a wider range of patients.

## V. Conclusion

The MOO method described here was applied for breast and OAR imaging in the chest wall. Our MOO approach is adaptable and can be applied to any coil shape and/or any imaging requirements. This method enabled us to achieve a 10 cm deep view of the OAR *in vivo*, which is consistent with the requirements for PT. Of note, this RF coil optimization method can be used to accommodate higher resonance frequencies.

We have demonstrated that breast imaging is feasible at ULF. However, the imaging requirements for PT require higher spatial and temporal resolution. Operation at higher field strengths (around 50 mT) but still in the low-field regime, and with higher gradient strengths (around 10 mT/m) should meet those requirements. Indeed, the results shown here combined with the Monte Carlo simulations designed here support the design and construction of a future low field MR guided proton therapy system.

## Supplementary Material

supplement

This article has supplementary downloadable material available at https://doi.org/10.1109/TBME.2024.3520895, provided by the authors.

## Figures and Tables

**Fig. 1.**
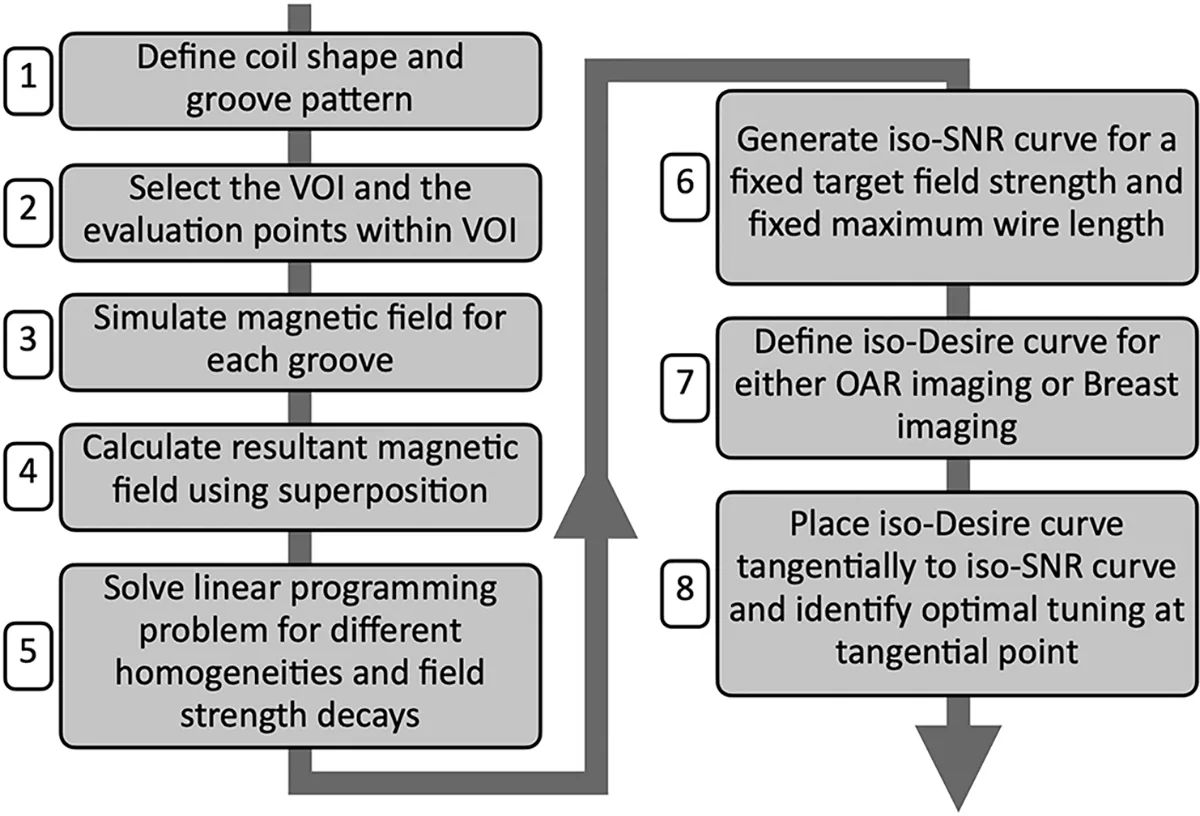
Optimization steps from the coil shape & groove pattern determination to the actual optimized groove occupation pattern obtained from the chosen iso-Desire line. VOI – volume of interest.

**Fig. 2.**
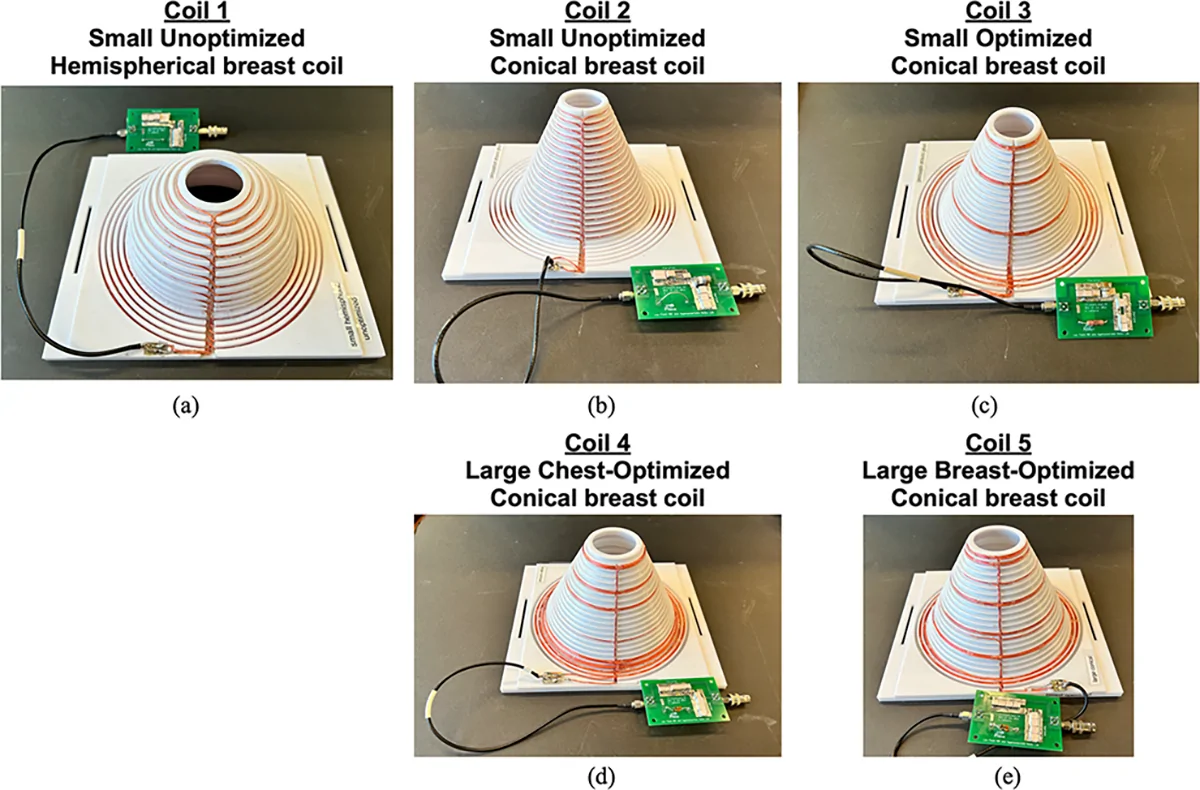
Photographs of the five breast coils - (a) Coil 1: the small unoptimized hemispherical coil, (b) Coil 2: the small unoptimized conical coil, (c) Coil 3: the small optimized conical coil, (d) Coil 4: the large chest-optimized conical coil and (e) Coil 5: the large breast-optimized conical coil.

**Fig. 3.**
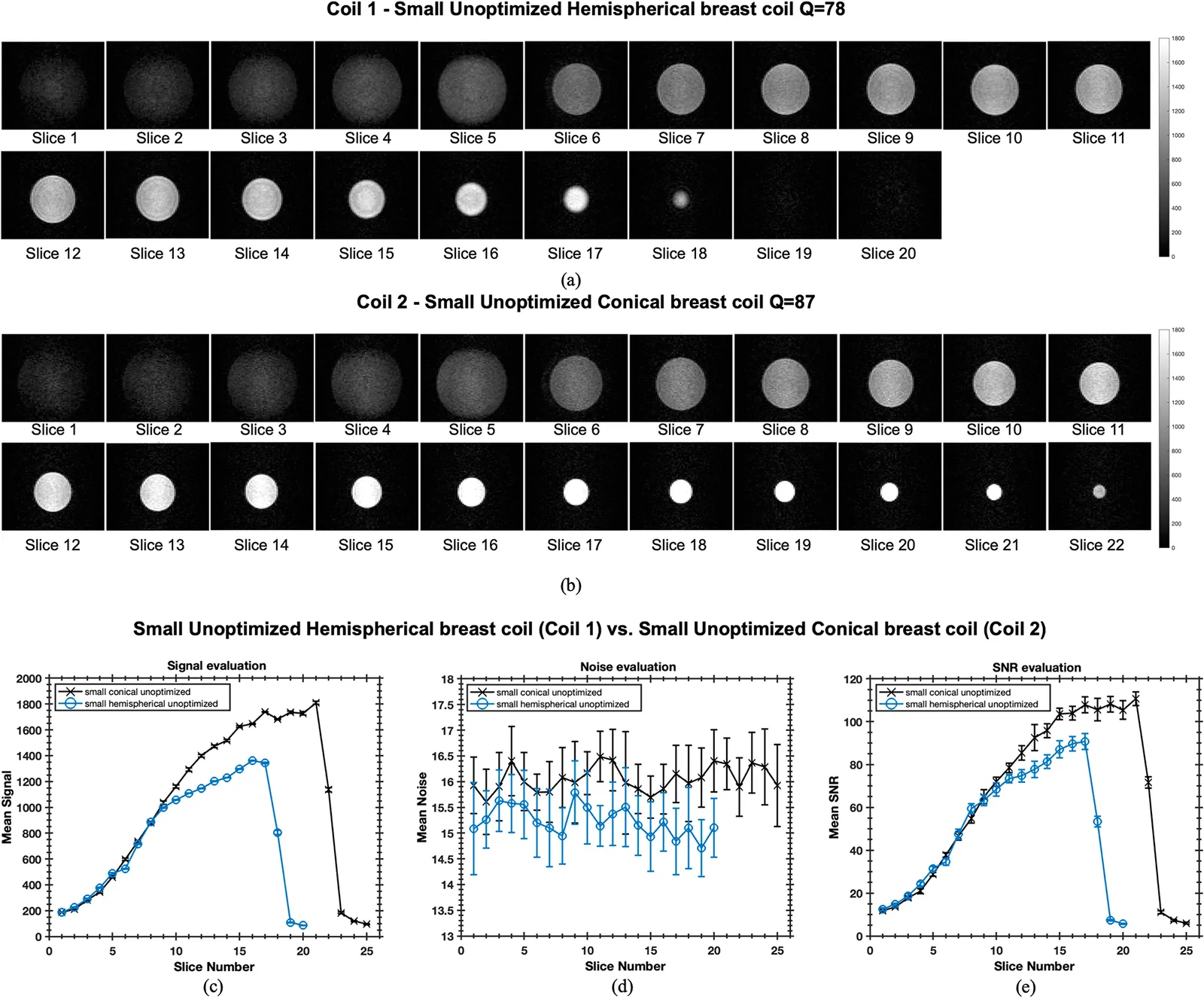
Phantom imaging experiments with small breast coils at 6.5 mT. (a) All 20 slices of the hemispherical coil (Coil 1) are displayed. (b) 22 slices out of 25 slices are shown for the small unoptimized conical coil (Coil 2). Slices 1 to 20/25 run through the posterior-to-anterior direction. The spatial resolution was the same across both coils. (c) Mean signal amplitude within the phantom, (d) Noise level measured in the background, and (e) the mean SNR were measured across the slices. For comparison, (c, d and e) represent the data for Coil 1 (ο - in blue) and for Coil 2 (× - in black). Each data point represents the mean and SD of 10 repeated experiments.

**Fig. 4.**
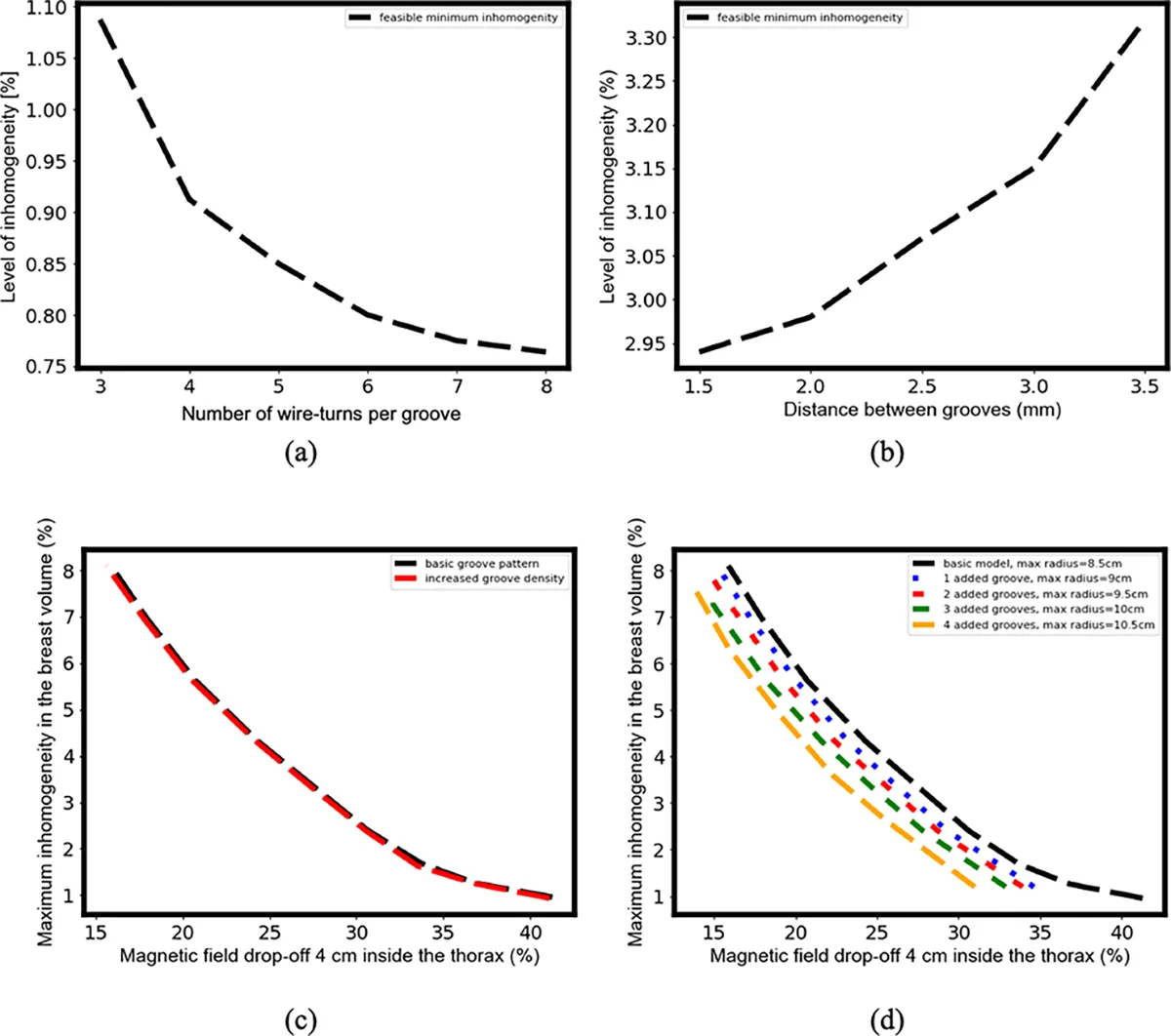
Groove Analysis – The influence of (a) the number of wire-turns per groove and (b) the groove distance within the breast volume on the attainable homogeneity level is shown, with all the other factors held constant. (c) Increasing the groove density (red dashed line) on the coil end plate does not much affect the resulting solution space. (d) Increasing the maximum groove radius on the coil end plate by adding additional grooves leads to a shift of the geometrically imposed solution space limitation. The values provided represent those along the coil’s main axis.

**Fig. 5.**
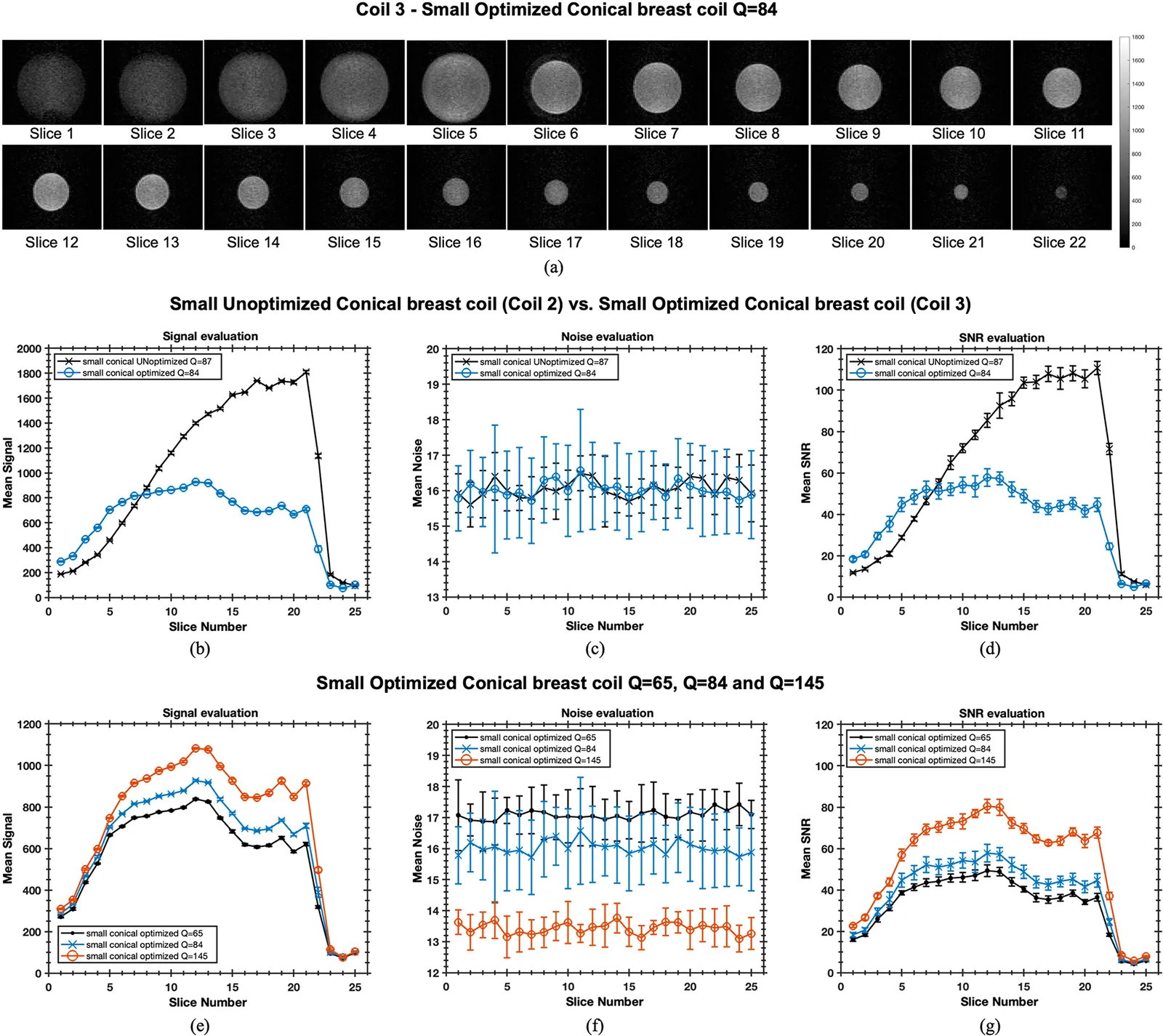
Phantom imaging experiments using Coil 3 at 6.5 mT and SNR analysis. (a) 22 slices out of 25 slices are shown for the small optimized conical coil – Coil 3 with a Q factor of 84. (b) The spatial resolution was the same across all coils. Slices 1 to 25 run through the posterior-to-anterior direction. (b and e) Mean signal amplitude within the phantom, (c and h) Noise level measured in the background, and (d and g) the mean SNR were measured across the slices. For comparison, (b, c, and d) represent the data for Coil 2 (× - in black) and for Coil 3 (ο - in blue), while (e, f, and g) represent the data for the optimized conical coil at Q factors of 65(• - in black), 84 (× - in blue) and 145 (ο - in orange). Each data point represents the mean and SD of 10 repeated experiments.

**Fig. 6.**
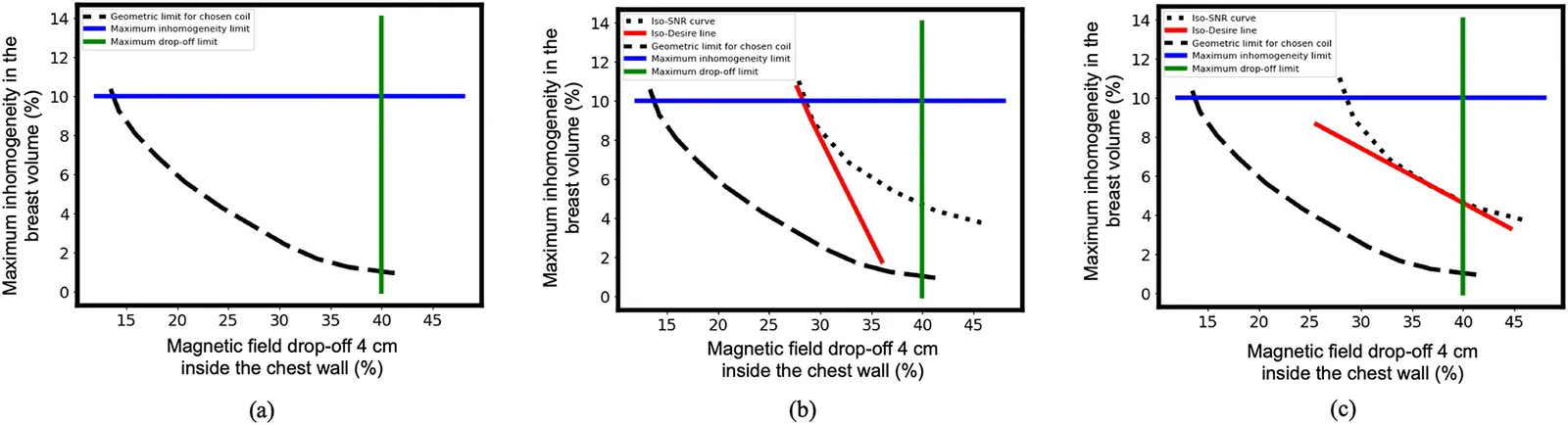
The solution space illustrated here for Coil 4, (a) is restricted by +/− 10% B_1_ inhomogeneity (blue line) and 40% B_1_ drop-off limits (green line) as well as by the geometric feasibility (dashed line). The geometric feasibility depends on the actual coil shape and the chosen groove pattern. The optimal spot is given as the tangential intersection of the iso-SNR curve with the iso-Desire curve. A steep iso-Desire line is represented in red in (b) and a flat iso-Desire line is represented in red in (c).

**Fig. 7.**
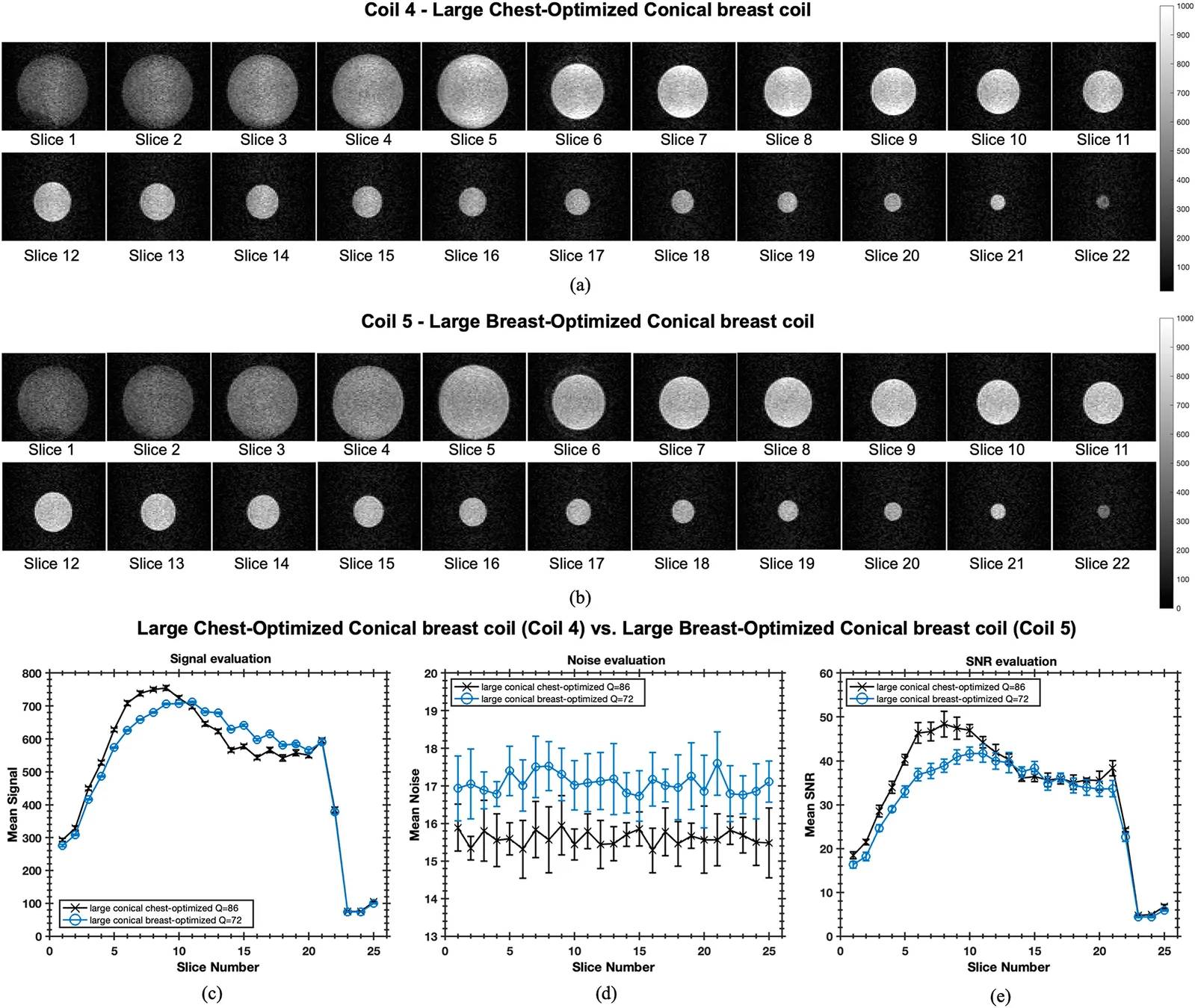
Phantom Imaging using either Coil 4, the chest-optimized breast coil (a) or Coil 5, the breast-optimized breast coil (b). 22 out of 25 slices are displayed for each coil. Slices 1 to 25 run through the posterior-to-anterior direction. The mean signal amplitude within the phantom region (c), the measured background noise level (d), and the mean SNR across all the slices (e) are plotted for Coil 4 (×) and Coil 5 (ο).

**Fig. 8.**
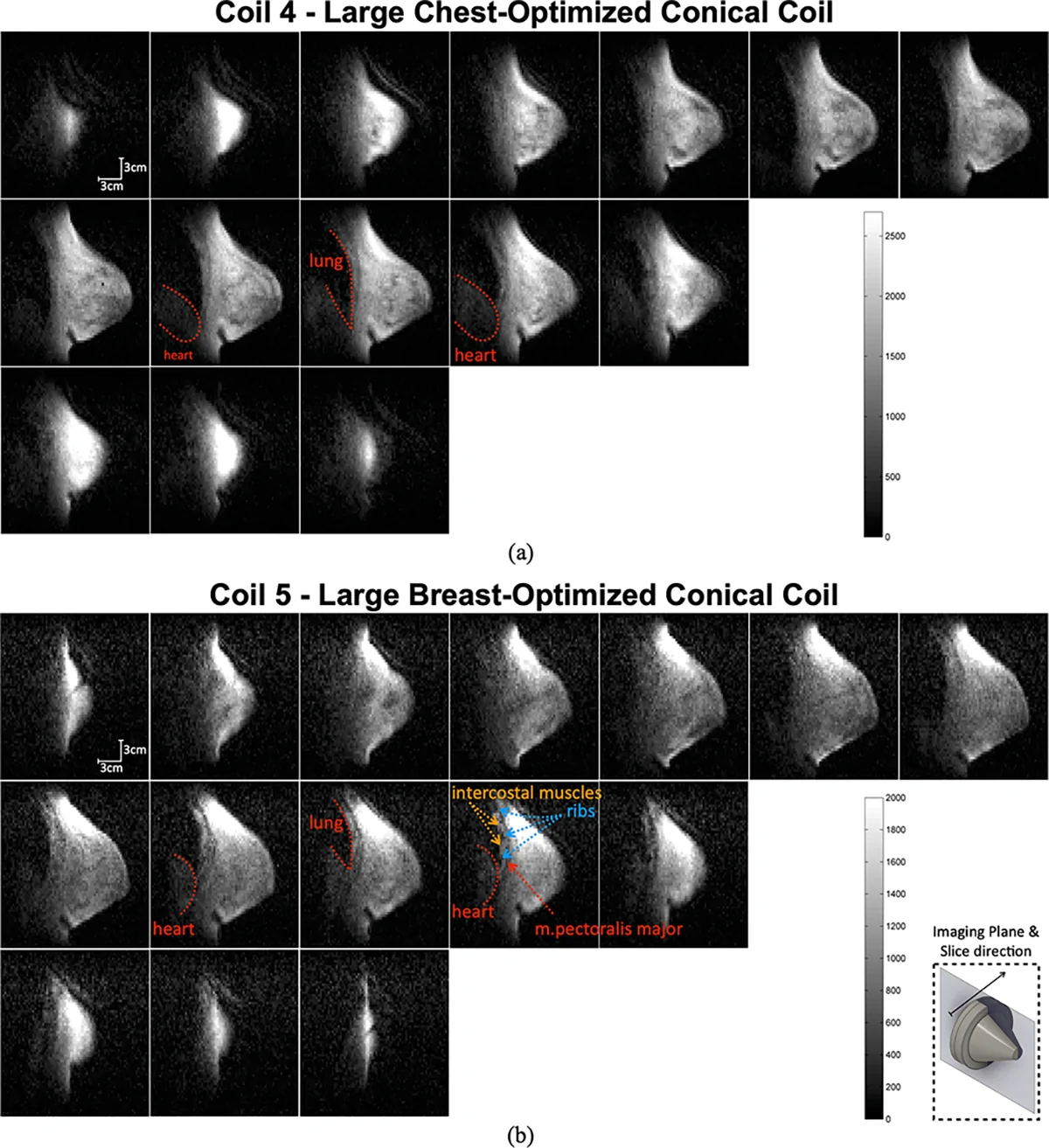
In vivo breast imaging on a female volunteer using Coil 4 and Coil 5. All 15 sagittal slices are shown with a spatial resolution of 3.5 mm × 2.5 mm × 7 mm for (a) the chest-optimized coil, Coil 4 and (b) the breast-optimized coil, Coil 5. The volunteer was imaged in an upright sitting position. The total scan time was 16 minutes and 45 seconds with 40 averages. Coil 4 provides a deeper view into the chest compared to Coil 5. The inset shows the imaging plane and the slice direction.

**Fig. 9.**
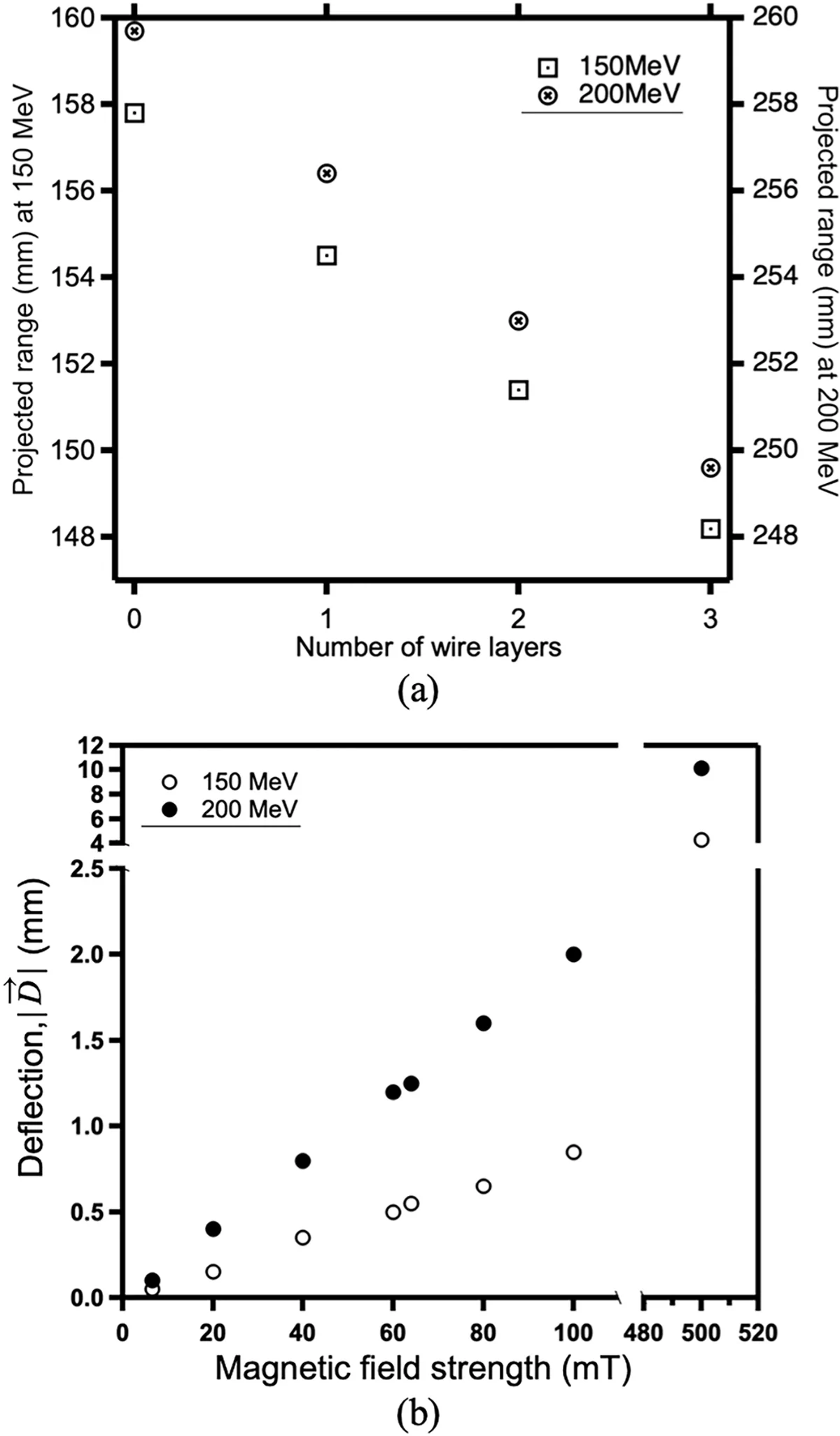
Wire influence on proton beam using Monte Carlo simulation – (a) Projected range of protons in water after passing through 0 up to 3 layers of copper wire for an initial proton energy of either 150 MeV (on the left y-axis) or 200 MeV (on the right y-axis). (b) Total Bragg peak deflection in a water tank depending on applied magnetic field strength with an initial proton energy of either 150 MeV or 200 MeV.

**TABLE I:** Properties of the Five Breast Coils

Coil #, Coil name	Volume (cm^3^)	Wire length (mm)	Inductance (μH)	Q factor	Resistor Added (Ω)
1, Small hemispherical unoptimized coil	598	655	150	78	0
2, Small conical unoptimized coil	652	780	160	88	0
3, Small conical optimized coil	652	1068	175	84	0.5
4, Large conical chest- optimized coil	1077	1591	230	86	2.1
5, Large conical breast-optimized coil	1077	1879	250	90	1.5

**TABLE II:** Groove Occupation Patterns for the Breast Coils

Coil #, Coil name	Groove Occupation Pattern	*B*_1_ inhomogeneity in the breast region (expressed as CoV)	*B*_1_ drop-off 4 cm inside chest wall (%)	Measured CoV for bSSFP phantom images (CoV +/− SD over 10 repeated scans)
1, Small hemispherical unoptimized coil	1,1,1,1,1,1,1,1,1,1,1,1,1,1,1,1,1,1	+/− 0.33	81	0.56 +/− 0.004
2, Small conical unoptimized coil	1,1,1,1,1,1,1,1,1,1,1,1,1,1,1,1,1,1,1,1,1,1,1,1	+/− 0.31	78	0.60 +/− 0.005
3, Small conical optimized coil	2,0,0,0,0,2,0,0,0,0,0,0,0,4,0,0,0,0,0,1,3,5,4,0	+/− 0.14	58	0.28 +/− 0.002
4, Large conical chest-optimized coil	0,5,0,0,0,2,0,0,2,0,0,0,0,0,1,0,0,0,5,6,10,10,0	+/− 0.16	38	0.22 +/− 0.002
5, Large conical breast-optimized coil	3,2,0,0,1,0,0,3,0,0,0,0,2,1,0,0,6,0,0,0,10,9,0	+/− 0.17	67	0.21 +/− 0.001
