# Supplementary material for: Breast Coil Optimization for Low Field MRI and Future MR-Guided Proton Therapy
Source: IEEE Trans Biomed Eng. Author manuscript; Available in PMC 2026 Nov 7. (PMC13744145; doi:10.1109/TBME.2024.3520895)
Supplement: Supplementary file 1 [file NIHMS2217359-supplement-supplement.pdf]

Supplementary Material (IEEE - TBME)

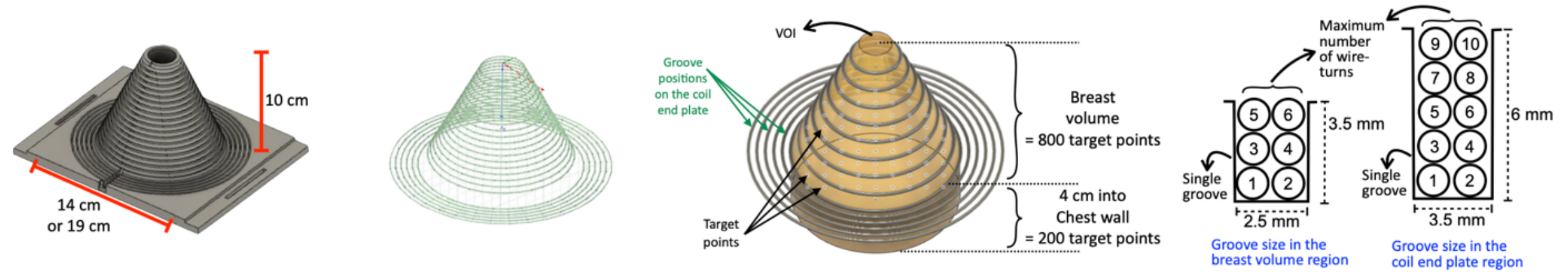

(a) CAD model (b) FEM simulation of the coil (c) Sketch of the mathematical model (d) Cross-section of single groove

Figure S1: (a) Fusion 360 CAD model of a typical breast coil geometry. The coil consists of a conical breast region and an additional end plate. The grooves are arranged in a circular manner over the coil surface. (b) FEM (Ansys Maxwell) simulation of the coil. The individual wire grooves are represented in green. The contribution of each single groove with unit current to the magnetic field at 1000 target points in the VOI is determined. (c) 3D sketch of the mathematical model with wire groove positions are shown on the surface of the conical breast coil in grey, the chosen VOI for optimization is highlighted in yellow, and the dots represent a selection of the target points. (d) 2D sketches of the cross-section of a single groove with the maximum copper wire arrangement. Diameter of each copper wire is 0.55 mm. The dimensions of the groove in the breast volume region are 2.5 mm broad and 3.5 mm deep. The dimensions of the groove in the coil end plate region are 3.5 mm broad and 6 mm deep.

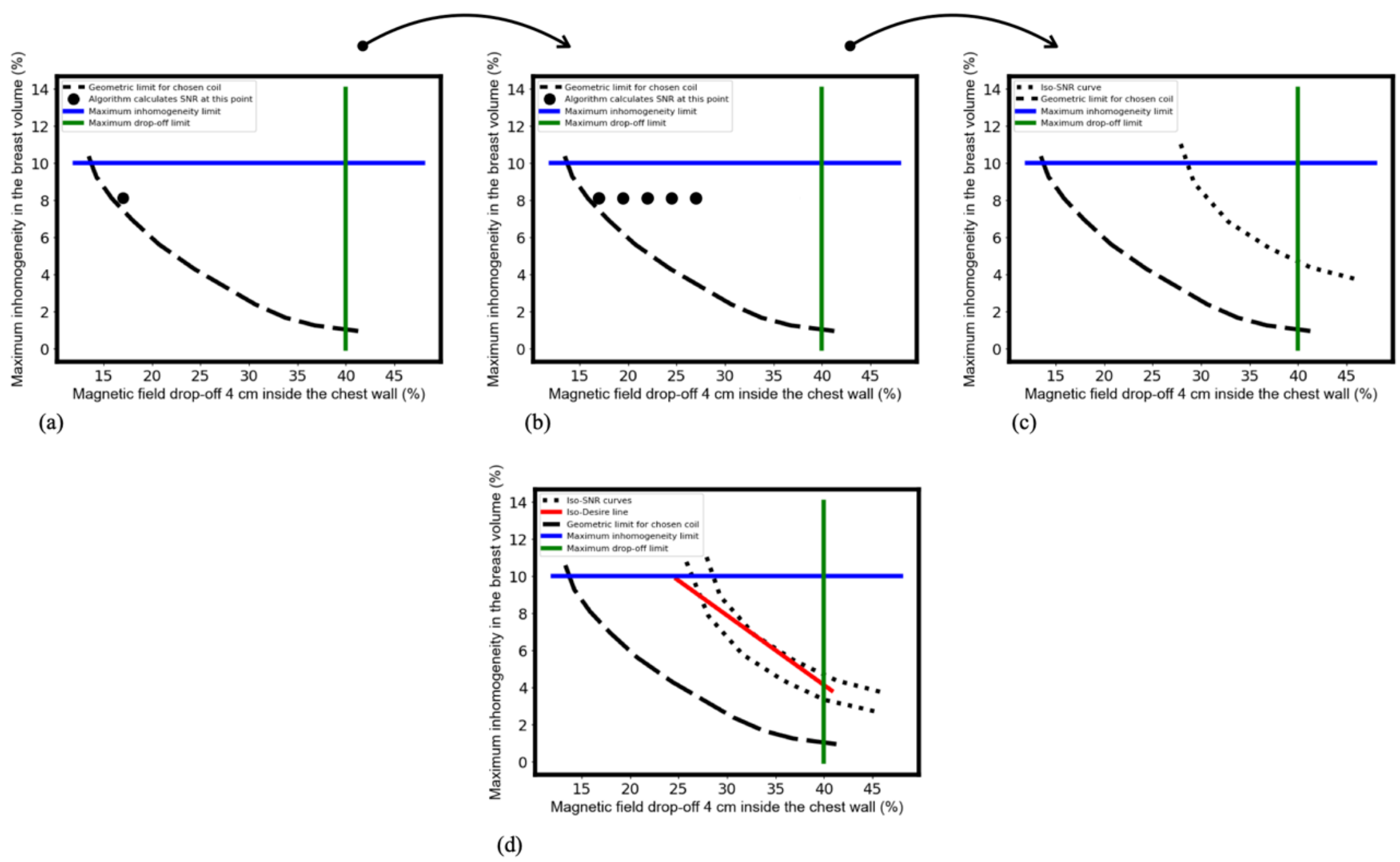

Figure S2: Illustration of the optimization steps – The solution space (a) is restricted by +/- 10 % B1 inhomogeneity (blue line) and 40 % B1 fall-off (green line) limits as well as by the geometric feasibility (dashed line). The algorithm scans the solution space step-by-step in incremental steps of 0.1 % and calculates the maximum SNR for each black spot. (b) Connecting spots with same SNR values results in the emerging of Iso-SNR curves. (c) Different SNR levels (from equation 12 in the main text) result in distinct iso-SNR curves. (d) For a calculated (or selected) SNR level k, the optimal tuning of the MRI coil for a specific imaging purpose is achieved at the point where the corresponding iso-SNR curve tangentially intersects the iso-Desire line (indifference curve).*

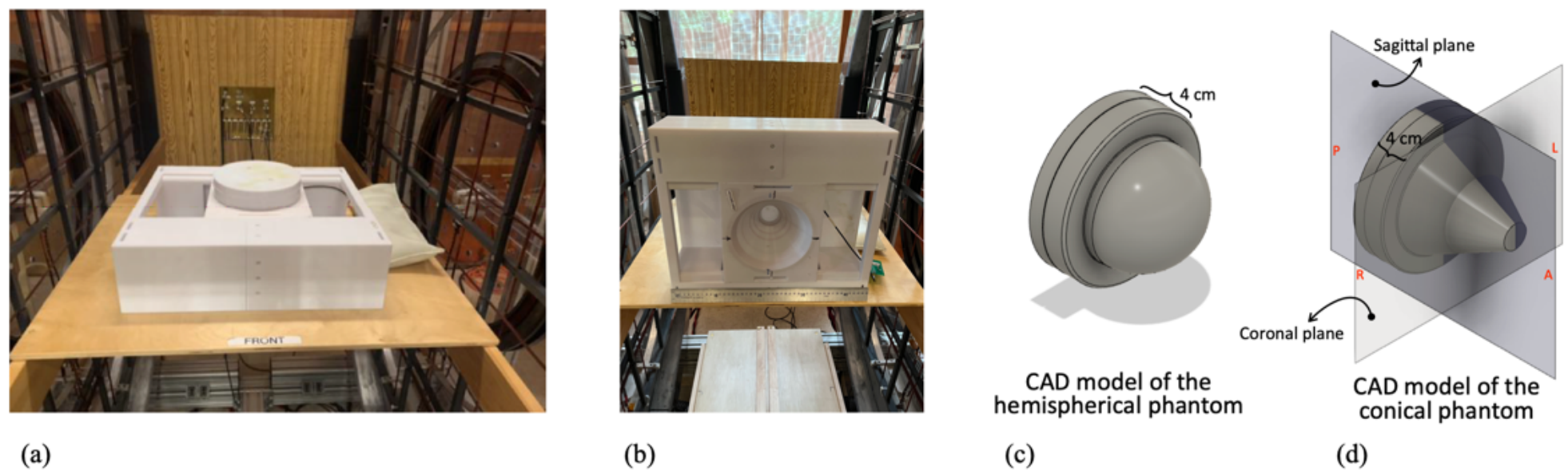

Figure S3: Imaging setup – (a) The phantom was placed in the custom-made support box which itself is placed on a height- adjustable board at the center of the MRI magnet. This coil produces a B1 field in the vertical direction, orthogonal to the B0 field which points left-to-right in this image. (b) For in vivo imaging, the support system was mounted upright on the height-adjustable board and positioned at the center of the magnet. The volunteer then sat on a chair in front of the box and positioned her breast into the coil. The support box allowed the volunteer to rest her arms, facilitating the placement of her chest as close as possible to the coil. (c) Fusion 360 CAD model of the hemispherical phantom including a 4 cm deep "chest wall" region. (d) Fusion 360 CAD model of the conical phantom with a 4 cm deep chest wall region. The coronal and sagittal planes used for imaging the phantom and two volunteers respectively are indicated.

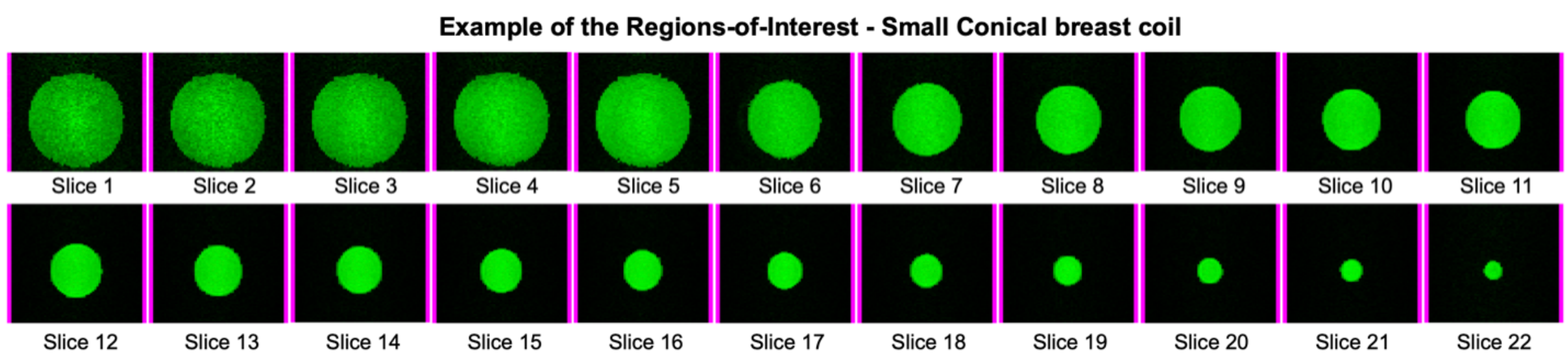

Figure S4: The regions-of-interest used for Signal-to-Noise ratio analysis. For each of the 22 slices, the region shaded in green was used to evaluate the mean signal amplitude and in each of the 22 slices, the regions shaded in magenta were used to calculate the standard deviation of the background noise. For noise evaluation, the identical regions were selected across all phantom imaging experiments to ensure a fair comparison.

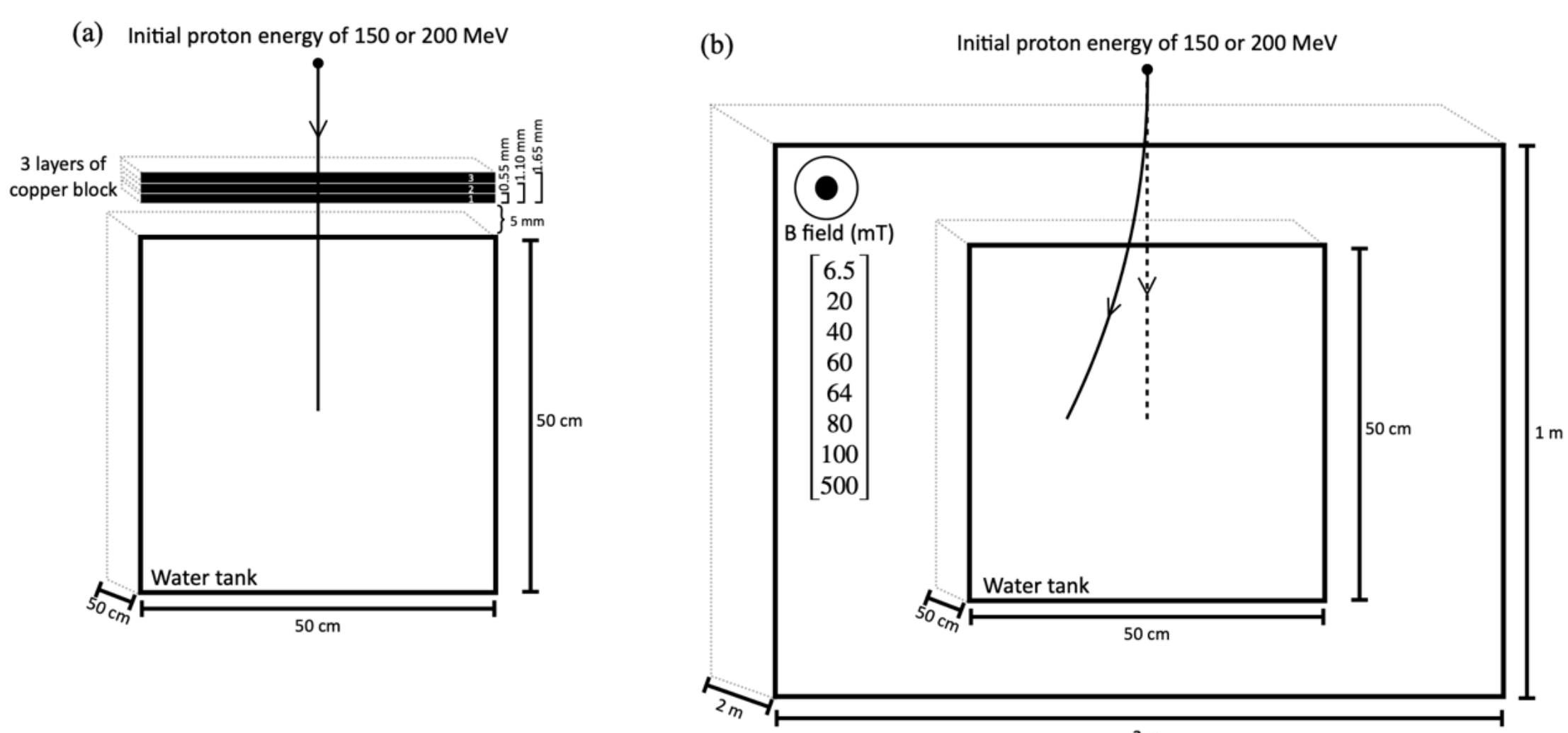

Figure S5: The experimental setups of the Monte Carlo Simulations of a low/ultra-low MRI system coupled to a proton beam. (a)The proton beam simulation setup to assess the influence of three layers of copper wire, each with a diameter of 0.55 mm, on the beam trajectory. A water tank of 50 cm × 50 cm × 50 cm was used as phantom. Two

different initial proton energies of 150 MeV and 200 MeV were used. (b) The proton beam simulation setup to assess the beam deflection in varying magnetic field strengths. A water tank of 50 cm × 50 cm × 50 cm was placed at the center of the magnetic field environment. Two different initial proton energies of 150 MeV and 200 MeV were used.

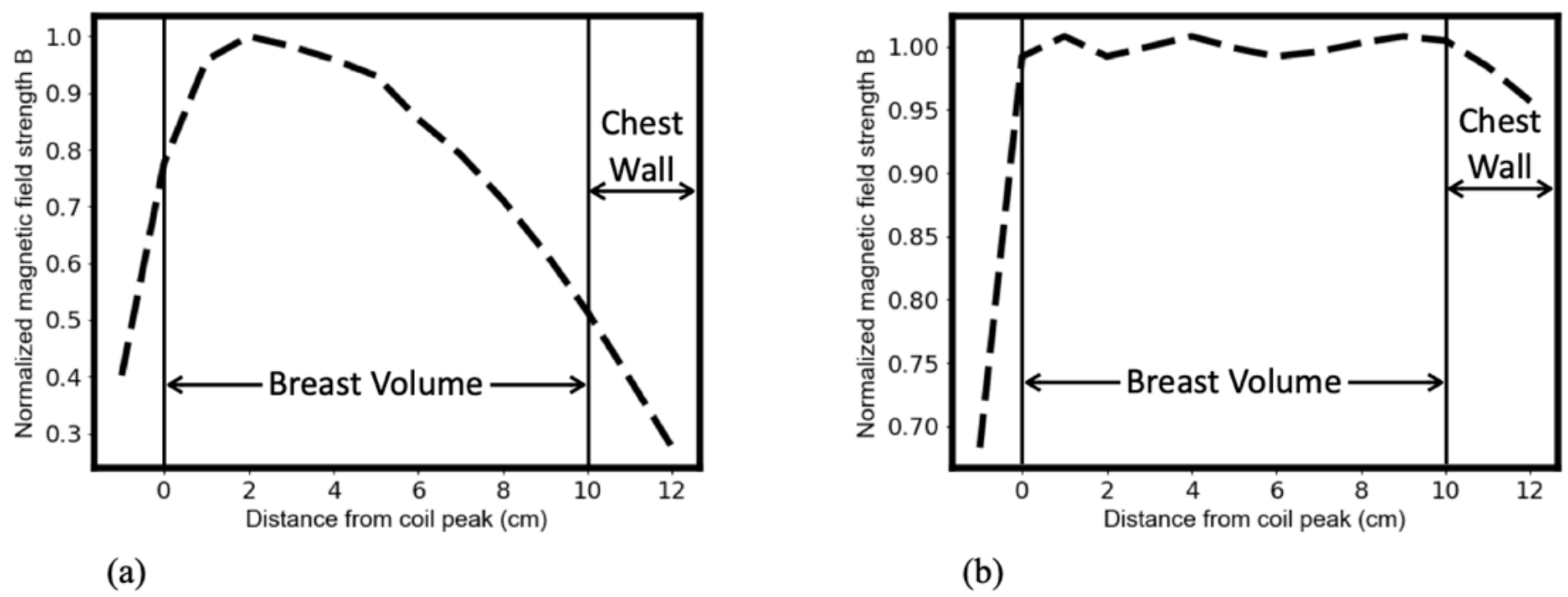

Figure S6: Comparison of the B_1 field homogeneity for (a) the small unoptimized conical coil, Coil 2, and (b) the small optimized conical coil, Coil 3. Finite Element Method simulations predict a more homogeneous magnetic field in the breast volume (± 18 % compared to ± 56 %) and a stronger signal in the chest wall part (62 % of the average signal strength in the breast compared to 31 %). An SNR analysis based on the total wire length of both coils predicted SNR values of the same order of magnitude.

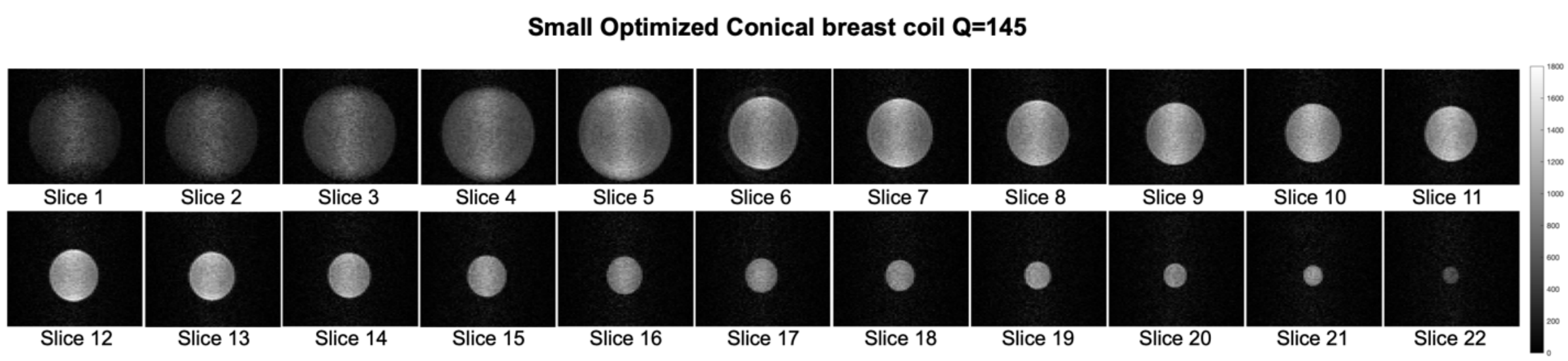

Figure S7: Phantom imaging experiments using the small optimized conical breast coil (Coil 3) with a Q factor of 145 at 6.5 mT.

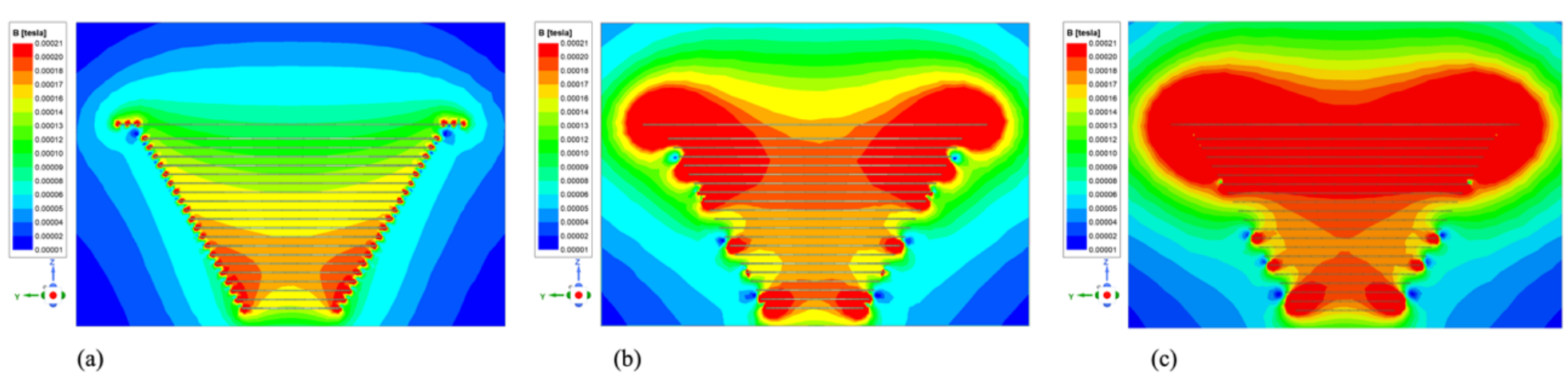

Figure S8: Simulated magnetic field strength for three breast coils of identical size and shape, each with different groove occupation patterns. (a) Large conical unoptimized coil was uniformly wound with one turn in each groove, both (b) the large conical breast-optimized coil, Coil 5, and (c) the large conical chest-optimized coil, Coil 4, were wound with the groove occupation patterns (shown in Table II of the main text).

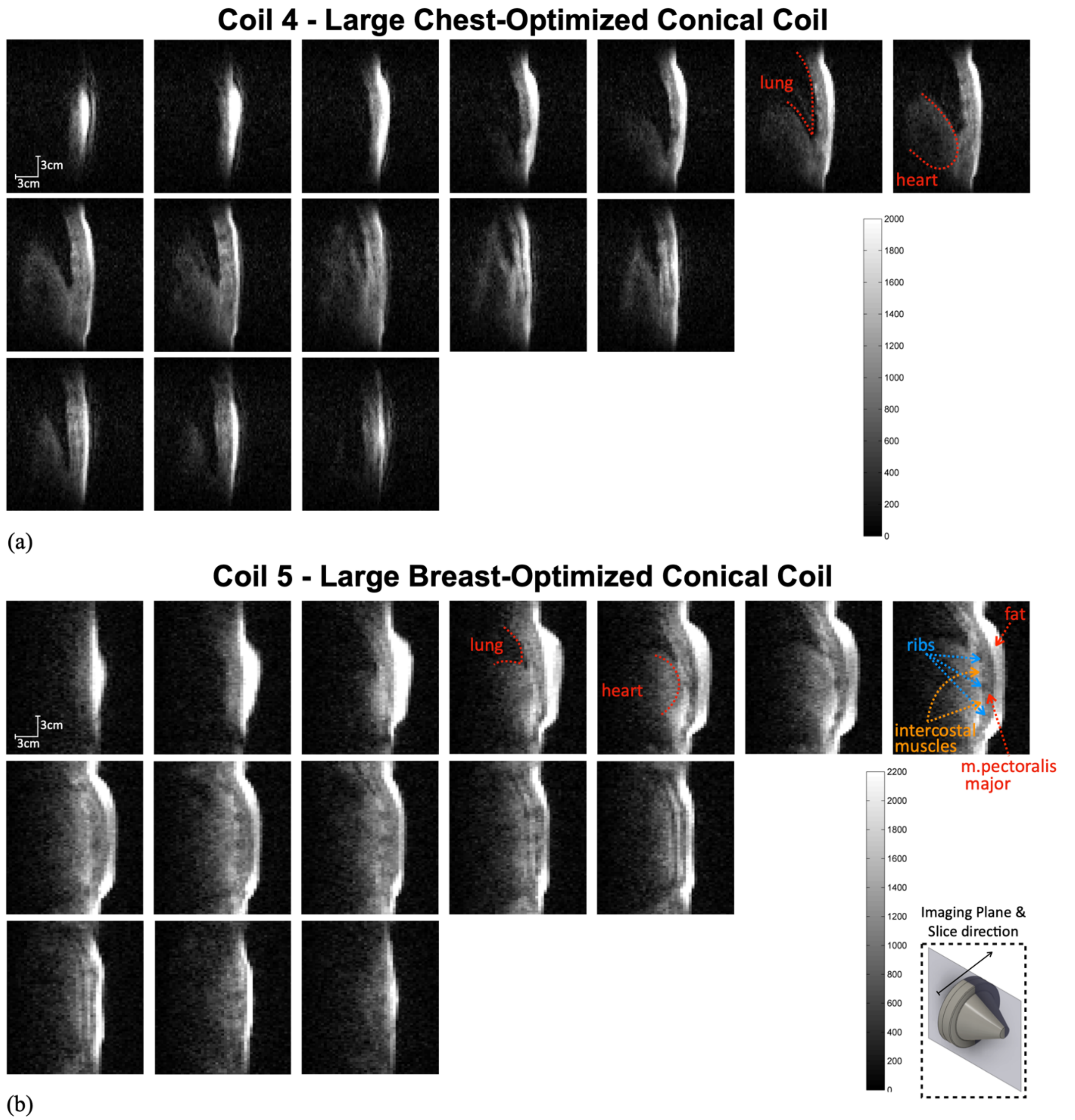

Figure S9: In vivo breast imaging on a male volunteer using large optimized conical coils. All the 15 sagittal slices are shown. Spatial resolution is 3.5 mm $\times$ 2.5 mm $\times$ 7 mm. Images shown were obtained using the chest-optimized coil, Coil 4, (a) and the breast-optimized coil, Coil 5 (b). The volunteer was imaged in an upright sitting position. The imaging plane and slice direction are shown in the inset at the bottom right corner. The total scan time was 16 minutes and 45 seconds with 40 averages. The chest-optimized coil provides a deeper view into the chest compared to the breast-optimized coil. The musculus pectoralis major, the ribs and the intercostal muscles, the anterior region of the lungs and the heart can be observed.
